# Two Goose-Type Lysozymes in *Mytilus galloprovincialis*: Possible Function Diversification and Adaptive Evolution

**DOI:** 10.1371/journal.pone.0045148

**Published:** 2012-09-21

**Authors:** Qing Wang, Linbao Zhang, Jianmin Zhao, Liping You, Huifeng Wu

**Affiliations:** Key Laboratory of Coastal Zone Environmental Processes, CAS, Shandong Provincial Key Laboratory of Coastal Zone Environmental Processes, Yantai Institute of Coastal Zone Research, Chinese Academy of Sciences, Yantai, PR China; University of Hyderabad, India

## Abstract

Two goose-type lysozymes (designated as MGgLYZ1 and MGgLYZ2) were identified from the mussel *Mytilus galloprovincialis*. MGgLYZ1 mRNA was widely expressed in the examined tissues and responded sensitively to bacterial challenge in hemocytes, while MGgLYZ2 mRNA was predominately expressed and performed its functions in hepatopancreas. However, immunolocalization analysis showed that both these lysozymes were expressed in all examined tissues with the exception of adductor muscle. Recombinant MGgLYZ1 and MGgLYZ2 could inhibit the growth of several Gram-positive and Gram-negative bacteria, and they both showed the highest activity against *Pseudomonas putida* with the minimum inhibitory concentration (MIC) of 0.95–1.91 µM and 1.20–2.40 µM, respectively. Protein sequences analysis revealed that MGgLYZ2 had lower isoelectric point and less protease cutting sites than MGgLYZ1. Recombinant MGgLYZ2 exhibited relative high activity at acidic pH of 4–5, while MGgLYZ1 have an optimum pH of 6. These results indicated MGgLYZ2 adapted to acidic environment and perhaps play an important role in digestion. Genomic structure analysis suggested that both MGgLYZ1 and MGgLYZ2 genes are composed of six exons with same length and five introns, indicating these genes were conserved and might originate from gene duplication during the evolution. Selection pressure analysis showed that MGgLYZ1 was under nearly neutral selection while MGgLYZ2 evolved under positive selection pressure with three positively selected amino acid residues (Y^102^, L^200^ and S^202^) detected in the mature peptide. All these findings suggested MGgLYZ2 perhaps served as a digestive lysozyme under positive selection pressure during the evolution while MGgLYZ1 was mainly involved in innate immune responses.

## Introduction

Lysozymes (EC 3.2.1.17) are antibacterial enzymes that can cleave the β-(1, 4)-glycosidic bond between N-acetylmuramic acid (NAM) and N-acetylglucosamine (NAG) in peptidoglycan layer of bacterial cell walls. It has been demonstrated that lysozymes are widely distributed in various organisms including bacteriophages, plants and animals. They are generally categorized into six types: chicken-type (c-type), goose-type (g-type), invertebrate-type (i-type), plant-type, bacterial-type and phage-type. The first three types of lysozymes have been found to exist in the animal kingdom [1]–[3].

Lysozymes have been found to play important roles in the immune system of the animals, especially in fish and invertebrates [4]–[7]. In addition, lysozyme serves as one of the major digestive enzymes in the true stomach of ruminants [8]–[9]. The functional diversification, biochemical adaption and the evolutionary mechanism of vertebrate c-type lysozymes have been well-studied [10]–[14]. For example, the molecular evolution of lysozyme from a defense to a digestive function has been evidenced in langur ruminant monkeys [9], the bird hoatzin [15] and the flies [16]–[17]. Recently, the adaptive evolution of i-type lysozyme from host defense to digestion had also been reported in oyster *Crassostrea virginica* [18].

The g-type lysozyme (gLYZ) was initially identified in geese egg white [19]–[21], and was later found to exist in fishes, mammals, urochordates and invertebrates [2], [22]–[25]. Most of the investigated animals were reported to possess only one type of gLYZ. Recently, multiple gLYZ genes had been reported in mouse and human, zebra fish, larvacean *Oikopleura dioica* and gastropod *Oncomelania hupensis* [22]–[23], [25]. Nelson et al. speculated that the multiple gLYZs in urochordates had been specialized, and the biochemically significant wide range of pI of the gLYZs in these animals may support this assumption [23]. In human, gLYZ1 mRNA was expressed only in kidney, while gLYZ2 transcript was detected mainly in eye and testis, indicating the specialization of gLYZs [22], [26]. However, no studies on the functional differentiation of gLYZs have been reported so far.

Bivalves are filter feeders constantly exposed to large amounts of bacteria present in their surrounding aqueous milieu. It has been demonstrated that bivalves can use bacteria as food sources to fulfill their nutritional requirement [27]. Thus, bivalves are thought to evolve strong capacity to hydrolyze bacteria [28], and lysozymes have been suggested to possess digestive capability in addition to bactericidal effect in bivalves [7], [18], [28]. In this study, the molecular characteristics, enzymatic properties, functions and evolutionary mechanism of two gLYZs obtained from *M. galloprovincialis* were investigated. The main objective of this study was to demonstrate the functional diversification between these two gLYZs and to test the role of positive selection during the evolution.

## Materials and Methods

### Animal culture

**Figure 1:**
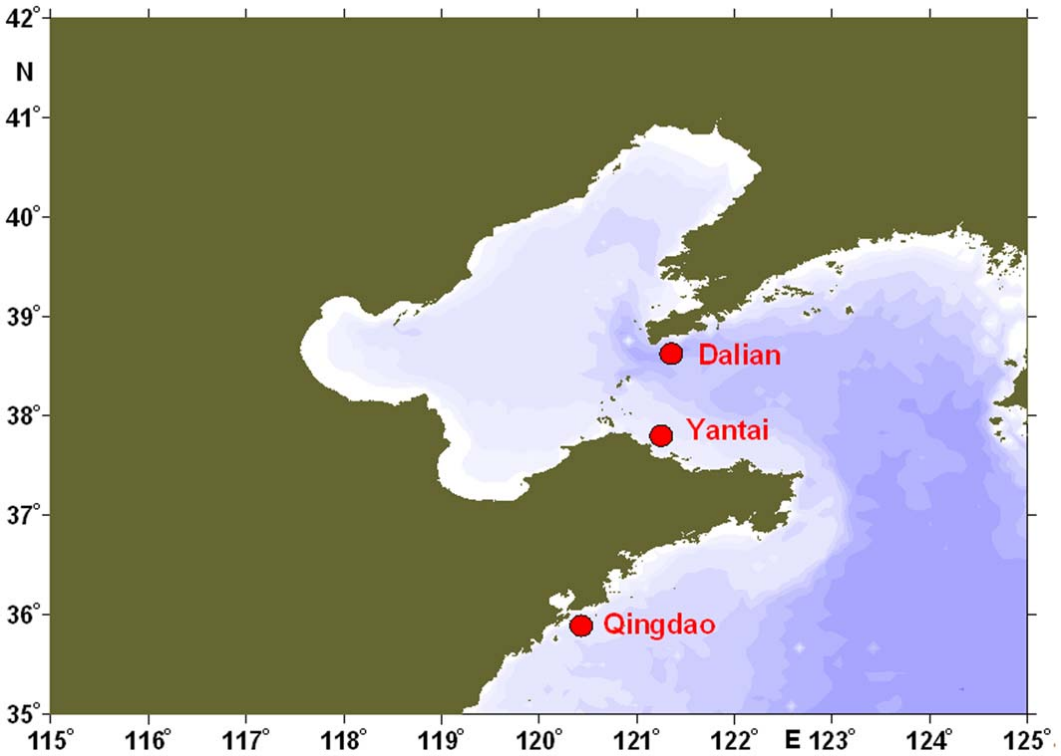
The sampling sites of *Mytilus galloprovincialis* along the coastal area of northern China.

Adult *M. galloprovincialis* (shell-length: 4.0–5.0 cm) were collected from a local culturing farm (Yantai, China, Figure 1) and then washed with fresh seawater. The mussels were acclimatized in aerated seawater (32 psu, pH = 8.1, DO>10 mg/L) at 20°C for 10 days before the commencement of the experiment. During the acclimatization period, the mussels were fed with *Isochrysis galbana* and *Platymonas helgolandica*, and the water was totally exchanged daily.

### Total RNA extraction and full-length cDNA cloning

The soft tissues of four individuals were quickly dissected and frozen in liquid nitrogen. Frozen tissues were pulverized under liquid nitrogen, and subjected to total RNA extraction by using TRIzol Reagent (Invitrogen, USA). The extracted RNA was then treated with RQ1 RNase-Free DNase (Promega, USA) to remove DNA contamination. Single-strand cDNA was synthesized from total RNA with M-MLV reverse transcriptase (Promega, USA).

Two *M. galloprovincialis* EST sequences homologous to *Chlamys farreri* goose-type lysozyme were identified from a bacterial infected hemocyte cDNA library by large-scale sequencing (unpublished). The 5′ and 3′ ends of these goose-type lysozymes were obtained by rapid amplification of cDNA ends using the SMART RACE cDNA Amplification Kit (Clontech, USA). The PCR programs were carried out at 94°C for 5 min, followed by 30 cycles of 94°C for 30 s, 56°C for 1 min, 72°C for 1 min and a final extension step at 72°C for 10 min. The PCR products were ligated into pMD-18T simple vector (Takara, Japan), and transformed into *Escherichia coli* Top 10 F′ competent cells. Three positive clones were subjected to sequence analysis on an ABI3730 Automated Sequencer (Applied Biosystem, USA).

### Identification of MGgLYZ1 and MGgLYZ2 gene structures

The genomic DNA was extracted from the adductor muscles of four mussels by using the Genomic DNA Purification Kit (Promega, USA). The intron sequences of MGgLYZ1 and MGgLYZ2 were amplified with five set of gene-specific primers (Table S1) based on each cDNA sequence, respectively.

### Bioinformatics analysis of MGgLYZ1 and MGgLYZ2 sequences

The searches for nucleotide and protein sequence similarities were performed with the BLAST algorithm (http://www.ncbi.nlm.nih.gov/blast). Multiple alignments were conducted with the ClustalW program (http://www.ebi.ac.uk/clustalw/). The deduced protein sequences were analyzed with ExPASy (http://www.expasy.org/). Signal peptide was predicted by SignalP 4.0 server (http://www.cbs.dtu.dk/services/SignalP/). Repeated gene sequences were identified by running the Tandem Repeats Finder program (http://tandem.bu.edu/trf/trf.html). Prediction of putative disulfide bonds was performed using the Dianna 1.1 web server (http://clavius.bc.edu/~clotelab/DiANNA/). The PSIPRED Protein Structure Prediction Server (http://bioinf.cs.ucl.ac.uk/psipred/) was used to predict the secondary structure. The three-dimensional structure of MGgLYZ1 and MGgLYZ2 were predicted by SWISSMODEL (http://swissmodel.expasy.org/workspace) based on the crystal structure of g-type lysozyme from Atlantic cod (PDB ID: 3mgw and 3gxr). The reliability of modeled structure was validated by Ramachandran plot analyses using PROCHECK (http://nihserver.mbi.ucla.edu/SAVES/). A phylogenetic tree of gLYZs was constructed with Mega4.1 software using the neighbor-joining (NJ) method. Bootstrap analysis was used with 1000 replicates to test the repeatability. Bayesian phylogenetic tree of mollusk gLYZs was generated from coding sequences with MrBayes 3.1.2. The optimal model of DNA sequence evolution was selected using jModelTest package 0.1.1.

### Test of evolution selection

For polymorphism detection in coding region of MGgLYZ1 and MGgLYZ2, a total of 18 adult *M. galloprovincialis* collected from three geographic origins (Qingdao, Yantai and Dalian; Figure 1) were used. During the experiment, the mussels were cultured as described above in the acclimatization period. No specific permits were required for the described field studies. The challenge experiment was conducted according to the method described by Schmitt et al [29]. For each geographic population, 50 µl live *Micrococcus luteus* (1×10^7^ CFU mL^−1^) and *V. anguillarum* (1×10^7^ CFU mL^−1^) were injected into the adductor muscle respectively. Then the hemocytes and hepatopancreas of six mussels from each geographic origin (total 18 individuals) with different treatment were sampled at 96 h post challenge. Total RNA from each tissue (total 72 samples) was immediately extracted and subjected to reverse transcription and PCR amplification. The PCR amplification was conducted with Pfu DNA polymerase. The primers used to amplify the coding regions were shown in Table S2. The PCR products were cloned into pMD-18T simple vector (Takara, Japan) and transformed into the competent cells of *E. coli* Top 10 F′. One positive clone of each sample was sequenced in both directions on an ABI3730 Automated Sequencer (Applied Biosystem, USA) by Chinese National Human Genome Center (SinoGenoMax). The coding regions of MGgLYZ1 (72 positive clones) and MGgLYZ2 (70 positive clones, two sample with no positive amplification) were sequenced respectively.

The nucleotide sequences encoding amino acids of the MGgLYZ1 (72 sequences) and MGgLYZ2 (70 sequences) were used to construct the NJ phylogeny trees with Kimura 2-parameter model respectively. The reliability of interior branches of each phylogeny was assessed with 1000 bootstraps. The phylogeny was used to estimate nonsynonymous to synonymous rate ratio (ω = dN/dS) by the maximum likelihood (ML) method implemented in CODEML program of the PAML 4.4 software package [30]. Positive selection can be inferred from a higher proportion of nonsynonymous than synonymous substitutions per site (dN/dS>1). Likelihood ratio tests (LRTs) were used to determine whether any codon positions were subjected to positive selection as indicated by ω>1.

To test for heterogeneous selective pressure at amino acid sites, the site-specific models were tested: M0 (one-ratio) against M3 (discrete), M1a (nearly neutral) against M2a (positive selection), M7 (beta) against M8 (beta & ω). The M0 (one-ratio) model assumes the same ω value for the entire tree. The M3 (discrete) model uses a general discrete distribution with three site classes, with the proportions p0, p1, and p2 and the ω ratios ω0, ω1, and ω2. The M1a model estimates single parameter, p0, with ω0 = 0, and the remaining sites with frequency p1 (p1 = 1–p0) assuming ω1 = 1. The M7 model assumed a beta distribution for the ω values between 0 and 1. M1a and M7 belong to null models that do not allow for any codons with ω>1, while M2a and M8 represent more general models that do. The LRTs between nested models were conducted by comparing twice the difference of the log-likelihood values (2ΔL) between two models with the χ^2^ distribution (df = 2). The Naive Empirical Bayes (NEB) method and Bayes empirical Bayes (BEB) method were used to calculate the posterior probabilities that each codon is from the site class of positive selection under models M3, M2a and M8 respectively [31].

### Tissue-specific expression of MGgLYZ1 and MGgLYZ2 mRNA

Hemocytes, gonad, gill, hepatopancreas, mantle and adductor muscle were taken aseptically from four mussels and subjected to total RNA extraction. Total RNA extraction and cDNA synthesis were performed as described above. qRT-PCR was carried out in an ABI 7500 Real-time Detection System by using the SYBR ExScript qRT-PCR Kit (Takara, Japan). The PCR amplification was carried out in a total volume of 50 µL, containing 25 µL of 2× SYBR Green PCR Master Mix, 20 µL of the diluted cDNA, 1 µL of each of primers (10 µmol/L), and 3 µL of DEPC-treated water. The thermal profile for qRT-PCR was 50°C for 2 min, 95°C for 10 min followed by 40 cycles of 95°C for 15 s and 60°C for 1 min. All reactions were run in triplicate. Dissociation curve analysis of amplicons was performed at the end of each PCR reaction to confirm that only one PCR product was amplified and detected. Moreover, each qRT-PCR product was purified and sequenced to verify the PCR specificity. The expression level of MGgLYZ1 and MGgLYZ2 was analyzed using 2^−ΔΔCT^ method with β-actin as the internal control [32]. The primers used to quantify the expression levels of MGgLYZ1 and MGgLYZ2 were listed in Table S2.

### Temporal expression profile of MGgLYZ1 and MGgLYZ2 mRNA in hemocytes and hepatopancreas after bacterial challenge

After the acclimatization, the mussels were randomly divided into 6 groups and cultured in 20 L aquarium tanks, each containing 30 individuals. During the challenge experiment, the mussels were cultured as described above. For the bacterial challenge experiment, 50 µL of live *Vibrio anguillarum* resuspended in sterilized seawater (1×10^7^ CFU/mL) was injected into the adductor muscles of mussels in three tanks. The untreated three tanks were employed as the control groups. For each treatment, the hemolymph and hepatopancreas of four randomly selected individuals were sampled for total RNA extraction at 6, 12, 24, 48, 72 and 96 hours post challenge. After hemolymph collection, hemocytes were isolated by centrifugation for further RNA extraction. The RNA extraction, cDNA synthesis, qRT-PCR thermal profile and the data analysis were conducted as described above.

The normal distribution of the data was tested by Kolmogorov-Smirnov test and the homogeneity of variance of the data was tested by Levene's test with SPSS 16.0 software (SPSS Inc., USA). All data were analyzed by one-way analysis of variance (one-way ANOVA) using SPSS 16.0 software. *P*<0.05 was accepted as statistically significant.

### Recombinant expression, protein purification and identification

The nucleotide sequences encoding the mature peptides of MGgLYZ1 and MGgLYZ2 were cloned into pET-21a(+) vector (Novagen, Germany) and expressed in *E. coli* BL21 pLysS (DE3) (Novagen, Germany). The recombinant proteins were expressed as inclusion bodies and purified by HisTrap Chelating Columns (GE Healthcare, USA) under denatured condition (8 mol L^−1^ urea).

After SDS-PAGE, the target protein band was excised from the gel and cut into small pieces. The gels were processed and analyzed by the method described by Jiang et al. [33]. The MS/MS spectra were searched with BioWorks 3.1 software, using the SEQUEST algorithm.

### Preparation of polyclonal antibodies and western blotting analysis

The polyclonal antibodies of MGgLYZ1 and MGgLYZ2 were produced according to the method described by Yue et al [34]. Two male adult rabbits (purchased from the Qingdao Institute for Drug Control, China) were immunized by an initial multipoint subcutaneous injection of 0.5 mg purified recombinant proteins emulsified in complete Freund's adjuvant per rabbit. Thirty days after the initial injection, the rabbits received 4 injections of the same dose antigen emulsified in incomplete Freund's adjuvant, with a 10-day interval between the injections. One month after the last injection, the immunized rabbits were bled and the serum IgGs were purified by affinity chromatography using Protein A Sepharose column (GE healthcare, USA). The study protocol for the experimental use of the animals was approved by the Ethics Committee of National Center for Clinical Laboratories.

The specificity of antibodies was tested by western blot analysis. After SDS-PAGE, the samples were transferred onto PVDF membranes by electroblotting at 100 V for 1 h. The membranes were blocked with 5% non-fat dry milk containing 1% BSA at 37°C for 1 h, and then incubated with diluted rabbit anti-MGgLYZ1 (1∶5000) and anti-MGgLYZ2 (1∶10000) IgG antibodies in PBS at 37°C for 1 h. Then the membranes were washed three times with PBS containing 0.05% Tween-20 (PBS-T) and incubated with a 1∶2000 diluted horseradish peroxidase-conjugated goat anti-rabbit IgG (Huabio, China) in PBS at 37°C for 1 h. After washing three times with PBS-T, the protein bands on the membrane were detected with HRP-DAB detection kit (Tiangen, China).

### Immunohistochemistrical localization of MGgLYZ1 and MGgLYZ2 in different tissues

Four mussels were dissected after acclimatization in aerated seawater (32 psu, pH = 8.1, DO>10 mg/L) at 20°C for 10 days to sample the tissues. The slides of hemocytes were prepared according to the method described by Yang et al [35]. The gill, mantle, adductor muscle and hepatopancreas of mussels were dissected and fixed in paraformaldehyde (4%) solution, dehydrated in ethanol, embedded in paraffin and sectioned at 6 µm. Twelve slides (3 slides for each individual) were prepared for each sample type.

The slides of hemocytes and tissues were incubated with antibody of MGgLYZ1 and MGgLYZ2 (diluted 1∶5000 in PBS-T) at 37°C for 1 h in a moisture chamber. After three times washing with PBS-T, the slides were incubated at 37°C for 1 h with alkaline phosphatase-conjugated goat anti-rabbit IgG (Zhongshan, China) diluted at 1∶200 with PBS-T. The slides were washed three times with PBS-T, counter stained with haematoxylin, and mounted in buffered glycerin for observation. Rabbits' pre-immune serum was used as negative control. Positive signal was stained red and the other signals were stained blue.

### Lysozyme activity assay

The purified proteins were refolded in gradient urea-TBS glycerol buffer according to the method by Zhang et al [36]. Lysozyme activity was determined using the method described by Xue et al [6]. The assay was performed in a 96-well microplate, in which 20 µl sample were mixed with 180 µl *Micrococcus lysodeikticus* (0.8 mg ml^−1^, dissolved in 0.18 M ammonium acetate, pH 5.5) suspension. The absorbance of the plate was measured at 450 nm for 5 min with a Magellan plate reader (Tecan, Switzerland). All measurements of lysozyme activity were done in triplicate. The results were expressed as percent activity with the highest activity detected being defined as 100%. The concentration of purified protein was quantified by BCA method [37].

To determine the effect of pH and temperature on the activity of recombinant MGgLYZ1 and MGgLYZ2, a wide pH range (pH 4–10) and temperature range (10–60°C) was used. In addition, 10 µl (1.6 mg L^−1^) of periplasmic lysozyme inhibitor of gLYZ (PliG) purified from *E. coli* was added to 200 µl enzyme (final concentration, 0.03 mg L^−1^) and substrate mixture to test the inhibitory effect of PliG on these gLYZs.

### Antimicrobial activity of recombinant MGgLYZ1 (rMGgLYZ1) and MGgLYZ2 (rMGgLYZ2)

Antibacterial testing was carried out using two Gram-positive bacteria (*Staphylococcus aureus* and *Staphylococcus pasteuri*) and six Gram-negative bacteria (*Vibrio azureus*, *Vibrio Parahaemolyticus*, *Enterobacter cloacae*, *Enterobacter aerogenes*, *Pseudomonas putida*, and *Proteus mirabilis*). The minimum inhibitory concentration (MIC) was determined according to the method of Hancock (http://cmdr.ubc.ca/bobh/methods/). In sterile 96-well microtitre plates, the 12 columns of each row were filled with 0.1 ml sterilized Luria-Bertani (LB) broth. Dilute the LB broth in column 1 with 0.1 ml of recombinant proteins and blend together, and then do serial doubling dilutions in column 2 with an equal volume of liquid from column 1. Sequentially, columns 3–10 received 0.1 ml of a mixture of culture medium and recombinant proteins from previous column serially to create tested concentrations with 2-fold dilution, respectively. The tested strains were grown to exponential phase (OD_600_ = 1), and diluted with 1000 fold to give a concentration of 2–7×10^5^ CFU/ml. Then 10 µl of bacterial suspension was inoculated in each column from column 1 to column 11. Column 11 served as growth control, and column 12 served as blank control. The plates with *V. azureus* and *P. putida* were incubated at 28°C for 24 h, and the plates with other tested bacteria were incubated at 37°C for 24 hours. The absorbance of the plate was measured at 600 nm with a Magellan plate reader (Tecan, Switzerland).

The assay was done with triplicates and the MIC value was defined as the range between the highest concentration of the protein where bacterial growth was observed and the lowest concentration that caused 100% inhibition of bacterial growth.

## Results

### Sequence analysis of MGgLYZ1 and MGgLYZ2 cDNAs and genomic structures

The full-length cDNA sequences of MGgLYZ1 and MGgLYZ2 were deposited in GenBank under accession no. JQ244770 and JQ244771, respectively. Both nucleotide sequences of MGgLYZ1 and MGgLYZ2 were predicted to encode a polypeptide of 206 amino acid residues with the signal peptide comprising the first 16 residues. The mature peptide of MGgLYZ1 had a calculated molecular mass of 21.4 kDa and a pI of 8.21, while the mature peptide of MGgLYZ2 had a predicted molecular mass of 20.6 kDa and a pI of 7.04.

The determined MGgLYZ1 and MGgLYZ2 gene from the transcriptional start site to the transcriptional end site consisted of 6015 bp and 5870 bp respectively. Both MGgLYZ1 and MGgLYZ2 genes were composed of six exons (48, 66, 90, 114, 165 and 135 bp, respectively) and five introns. MGgLYZg1 had five introns with the length of 254, 273, 1905, 1320 and 1491 bp respectively, while the five introns of MGgLYZg2 were of 2065, 537, 946, 864 and 789 bp respectively (Figure S1).

Typical eukaryotic splice-sites were presented only in the fourth intron of MGgLYZ1 and MGgLYZ2 sequences at the exon–intron boundaries, including a GT at the 5′ boundary and an AG at its 3′ boundary. Repetitive elements such as the dispersed satellite repeats and one tandem repeat had been identified in the fourth intron of MGgLYZ1, and only three tandem repeats were found in the first intron of MGgLYZ2.

### Homology, structure prediction and phylogenetic analysis

**Figure 2:**
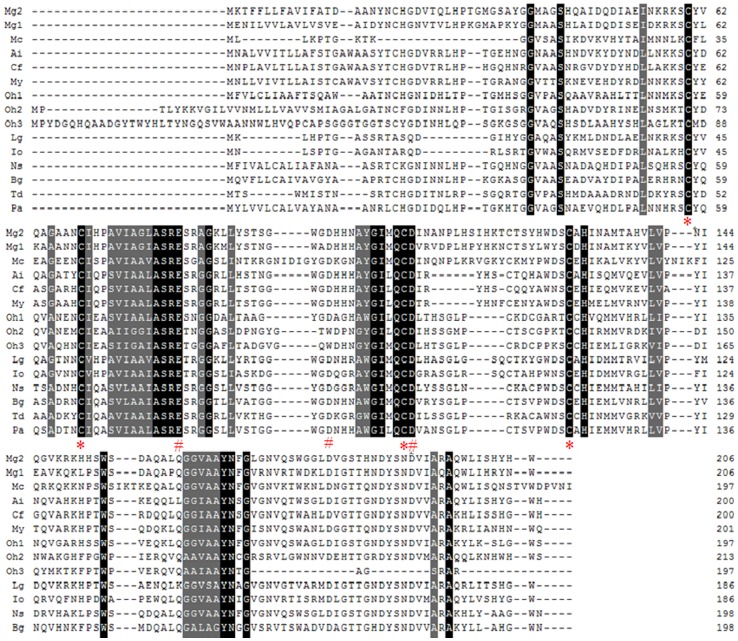
Multiple alignments of MGgLYZ1 and MGgLYZ2 with other mollusk gLYZs deposited in GenBank. The black shadow region indicates positions where all sequences share the same amino acid residue. Gaps are indicated by dashes to improve the alignment. The asterisk indicates the conserved cysteine residues and the pound sign indicates the conserved active residues in mollusk gLYZs. The GenBank accession no. and the species are as follows: JQ244770 (*Mytilus galloprovincialis* 1, Mg1), JQ244771 (*Mytilus galloprovincialis* 2, Mg2), DQ227696.1 (*Chlamys farreri*, Cf), AY788903 (*Argopecten irradians*, Ai), GR867752.1 (*Mizuhopecten yessoensis*, My), ES392226.1 (*Mytilus californianus*, Mc), FC670738.1 (*Lottia gigantean*, Lg), DC603639.1 (*Nesiohelix samarangae*, Ns), ES491677.1 (*Biomphalaria glabrata*, Bg), EV289297.1 (*Tritonia diomedea*, Td), ADV36303.1 (*Physella acuta*, Pa), FK716269.1 (*Ilyanassa obsolete*, Io), GW425811 (*Oncomelania hupensis* 1, Oh1), GW426148 (*Oncomelania hupensis* 2, Oh2) and GW427036 (*Oncomelania hupensis* 3, Oh3).

Multiple alignment of MGgLYZ1 and MGgLYZ2 with other mollusk counterparts (Figure 2) indicated that the amino acids critical for the fundamental structure and function of gLYZ were highly conserved in mollusk gLYZs, such as the substrate binding sites (Ile^104^, Ile^132^, Gly^163^, Gly^183^) and the catalytic residues (Glu^82^, Asp^97^, Asp^108^). Additionally, four cysteine residues (Cys^60^, Cys^69^, Cys^107^, Cys^129^) were totally conserved among the mollusk gLYZs.

**Figure 3:**
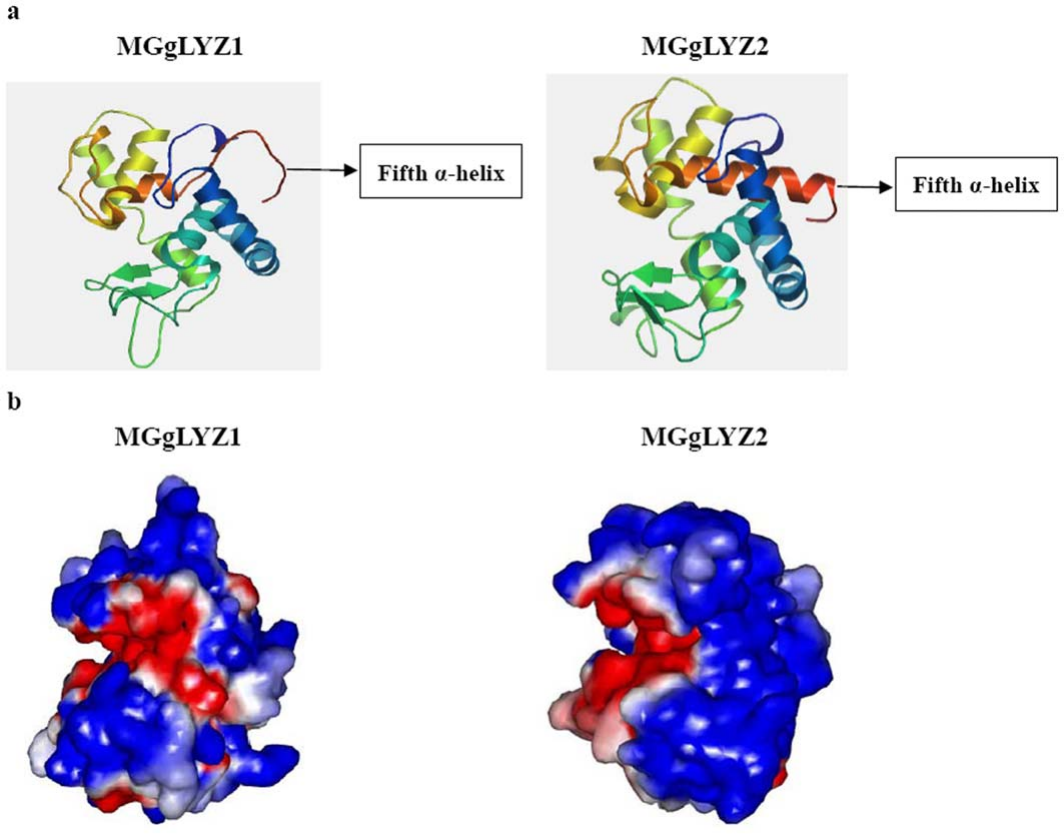
Predicted three-dimensional structure of MGgLYZ1 and MGgLYZ2. a: Three-dimensional structure predicted by SWISSMODEL. b: Electrostatic surface potentials of MGgLYZ1 and MGgLYZ2. The color runs from intense red (lowest) to intense blue (highest) potential.

It was suggested that two cysteines (Cys^53^-Cys^113^) of MGgLYZ2 might constitute a disulfide bond with a significant score (0.82117; Dianna 1.1), while no significant score for disulfide bond was obtained from MGgLYZ1. As predicted by PSIPRED Protein Structure Prediction Server, both MGgLYZ1 and MGgLYZ2 had five α-helixes and one β-sheet in their secondary structures. As shown in Figure 3a, the fifth α-helix in three-dimensional structure of MGgLYZ1 was different from that of MGgLYZ2. In addition, the electrostatic surface potential of MGgLYZ1 was also different from that of MGgLYZ2 (Figure 3b). According to Ramachandran plot, 84.0% residues of MGgLYZ1 lie in the most favored regions, 10.4% in the additionally allowed regions, and 1.8% residues in the disallowed regions. For MGgLYZ2, 88.1% residues lie in the most favored regions, 8.8% residues in the additionally allowed regions and 1.3% residues in the disallowed regions. The generated model of MGgLYZ1 and MGgLYZ2 seemed to be reliable with the good Ramachandran plot values with G-factors of −0.12 and 0.03, respectively [38].

**Figure 4:**
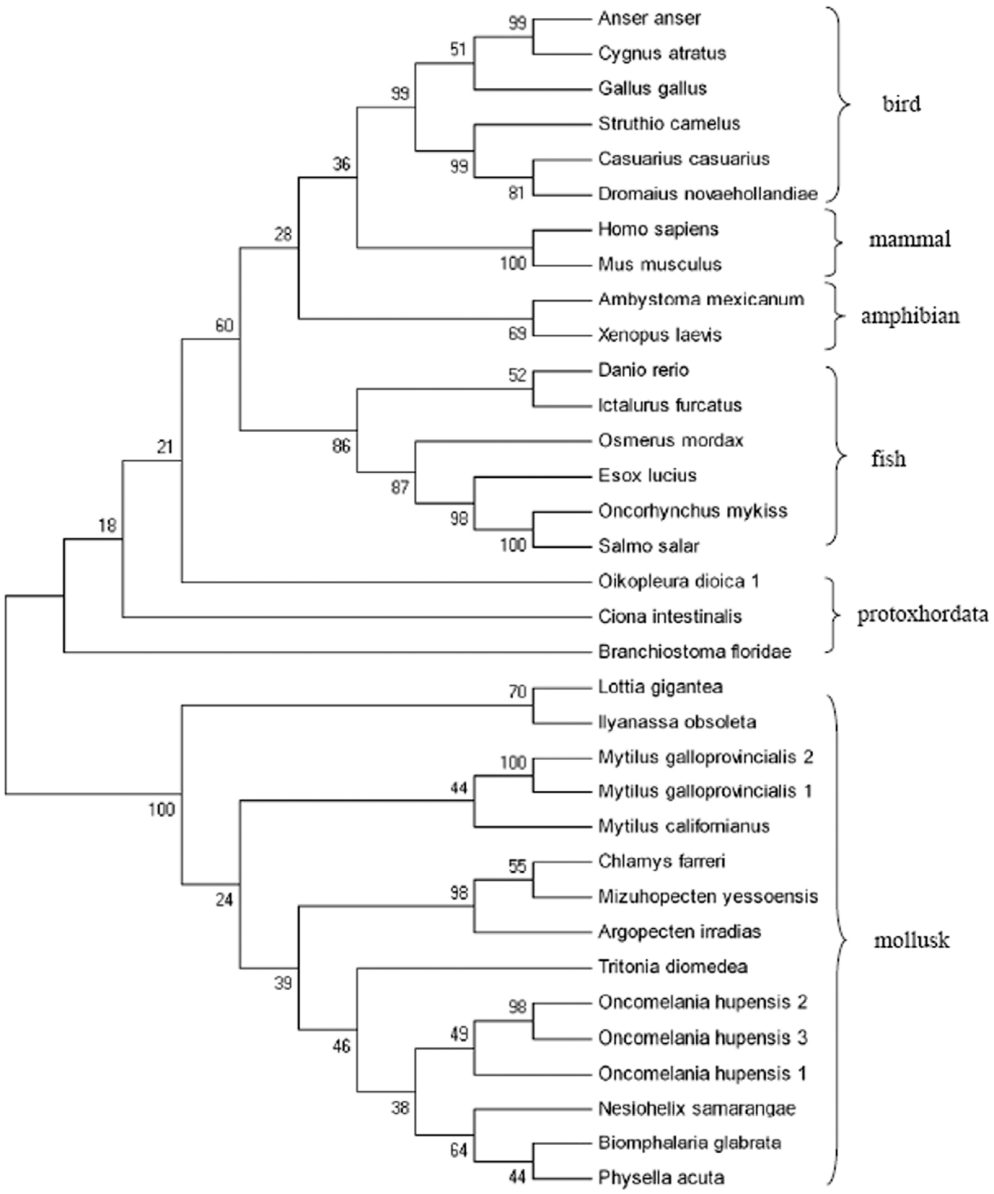
Phylogenetic tree constructed by neighbour-joining method based on the sequences of gLYZs from different animals. Numbers at the forks indicate the bootstrap values (in %) out of 1000 replicates. The sequences used to construct phylogeny trees of gLYZs are shown in Table S3.

Blast analysis revealed that gLYZ genes were widely detected in mollusks, from terrestrial species to freshwater and marine species (Table S3). In the phylogenetic tree of gLYZs (Figure 4), MGgLYZ1 and MGgLYZ2 were first clustered with gLYZ from the mussel *M. californianus*, and then clustered with scallop gLYZs, and formed an invertebrate (mollusk) sister group to the protoxhordata gLYZs and further grouped with gLYZs from vertebrates. The vertebrate clade possessed a mammalian, an avian, an amphibian and a fish subclade. The relationships revealed in the phylogenetic tree were in agreement with the concept of traditional taxonomy. According to the Bayesian tree of mollusk gLYZs (Figure S2), the evolutionary relationship of these mollusk gLYZs was similar to that of the NJ tree.

### Analysis of adaptive sequence evolution

The sequences of MGgLYZ1 have been deposited in the GenBank database under the accession numbers of JX184998–JX185069, and the accession numbers of MGgLYZ2 sequences were JX184928–JX184997. To test positive selection of MGgLYZ1 and MGgLYZ2, six models (M0, M1a, M2a, M3, M7 and M8) were implemented to construct the tree LRTs. The log-likelihood values and parameter estimates of the MGgLYZ1 and MGgLYZ2 under various site models were shown in Table 1.

**Table 1:** Evolutionary analysis of MGgLYZ1 and MGgLYZ2 genes.

Gene	Model	lnL	Estimates of parameters	2Δl	*P* value	Positively selected sites
MGgLYZ1	M0	−1149	ω = 1.27997	2	*P*>0.05	—
	M3	−1148	p0 = 0.54424, p1 = 0.44486, (p2 = 0.01090), ω0 = 1.14654, ω1 = 1.14655, (ω2 = 16.90789)			All of the amino acids
	M1a	−1149	P0 = 0.00001, (p1 = 0.99999)	2	*P*>0.05	Not allowed
	M2a	−1148	p0 = 0.43345, p1 = 0.55448, (p2 = 0.01208), ω0 = 1.0000, (ω1 = 1), ω2 = 14.74815			116P, 203R
	M7	−1149	p = 0.95401, q = 0.00500	2	*P*>0.05	Not allowed
	M8	−1148	p0 = 0.98792, p = 0.20022, q = 0.00500, (p1 = 0.01208), ω = 14.74799			11L, 20N, 27V, 31K, 33M, 44H, 48D, 49Q, 54I, 63K, 67N, 82E, 84R, 86G, 87K, 91S, 99H, 106Q, 109V, 112D, 116P, 133N, 135M, 148K, 156D, 173N, 179K, 183G, 193V, 202H, 203R
MGgLYZ2	M0	−1319	ω = 0.55040	18	*P*<0.01	—
	M3	−1310	p0 = 0.00037, p1 = 0.98079, (p2 = 0.01884), ω0 = 0.0000, ω1 = 0.42149, (ω2 = 13.32169)			5L, 102Y, 200L, 202S
	M1a	−1316	P0 = 0.55948, (p1 = 0.44052)	8	*P*<0.05	Not allowed
	M2a	−1312	p0 = 0.96687, p1 = 0.01620, (p2 = 0.01693), ω0 = 0.41968, (ω1 = 1), ω2 = 16.14571			5L, 102Y, 200L, 202S
	M7	−1317	p = 0.06414, q = 0.07120	14	*P*<0.001	Not allowed
	M8	−1310	p0 = 0.98176, p = 4.08597, q = 5.48325, (p1 = 0.01824), ω = 13.52309			5L, 102Y, 200L, 202S

The M0 model was compared with model M3 to determine the existence of dN/dS heterogeneity among the codons. For MGgLYZ1, the M0–M3 comparison revealed that M0 was better fit to the data (2Δl = 2, χ^2^ = 9.49, df = 4, *P*>0.05). In the M0 model, the ω value (ω = 1.27997) among the codons indicated all the amino acids of MGgLYZ1 were under weakly positive selection or nearly neutral selection. Although M2a model detected two sites and M8 model detected 31 positive sites (Table 1), M1a and M8 fit the data better than M2a and M7 as detected by LRTs respectively (*P*<0.05), suggesting all the amino acids of MGgLYZ1 detected by M1a vs. M2a and M7 vs. M8 were under nearly neutral selection. Thus, there was no amino acid in MGgLYZ1 under positive selection detected by these models.

For MGgLYZ2, LRTs gave significantly better results for M3 (2Δl = 18, χ^2^ = 13.2, df = 4, *P*<0.01), revealing significant variation in ω among the codons. The M3 model showed that approximately 0.04% of the codons have ω0 = 0.00, whereas 98.08% of codons have ω1 = 0.42, and only 1.88% of the codons have ω2 = 13.32. In addition, M2a fitted the data significantly better than M1a (2Δl = 8, χ^2^ = 5.99, df = 2, *P*<0.05), suggesting that 1.2% of the sites are under positive selection with ω2 = 16.1. Moreover, M8 fitted the data significantly better than M7 (2Δl = 14, χ^2^ = 13.8, df = 2, *P*<0.001), indicating 1.8% of the codons in MGgLYZ2 were under positive selection with ω = 13.5. Therefore, both Models M2a and M8 detected positive selection in MGgLYZ2. These analyses provided clearly statistical evidence to support an adaptive evolution event occurring in MGgLYZ2.

To identify the positive selection sites in MGgLYZ2, the NEB and BEB approaches were used to calculate the posterior probabilities of ω classes for each site. All selection models detected four identical positively selected sites (Leu^5^, Tyr^102^, Leu^200^ and Ser^202^), of which only Ser^202^ had a posterior probability greater than 0.99 (Table 1). In the secondary structure, the site Tyr^102^ was located in the β-strand of MGgLYZ2, while the sites Leu^200^ and Ser^202^ were located at the fifth α-helix (Figure 3a).

### Tissue expression profiles of MGgLYZ1 and MGgLYZ2 mRNA

**Figure 5:**
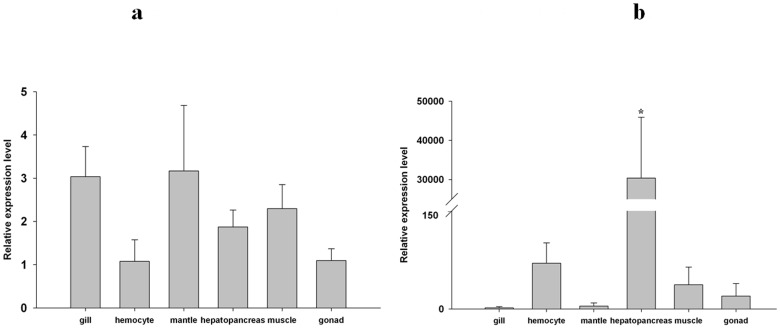
Tissue-specific expression of MGgLYZ1 (a) and MGgLYZ2 (b) mRNA. The mRNA expression level is calculated relative to actin expression and shown as mean ± SD (n = 4). Significant difference is indicated with an asterisk at *P*<0.05.

The tissue expression profiles of MGgLYZ1 (Figure 5a) and MGgLYZ2 (Figure 5b) were investigated by using real-time PCR technique. The transcript of MGgLYZ1 was widely detected in the tissues of gill, mantle, hepatopancreas, hemocytes and muscle, and there was no significant difference between these tissues. However, MGgLYZ2 transcript was dominantly expressed in hepatopancreas, and the expression level was significantly higher than that of other examined tissues (*P*<0.05).

### Temporal expression profiles of MGgLYZ1 and MGgLYZ2 mRNA in hemocytes and hepatopancreas after bacterial challenge

**Figure 6:**
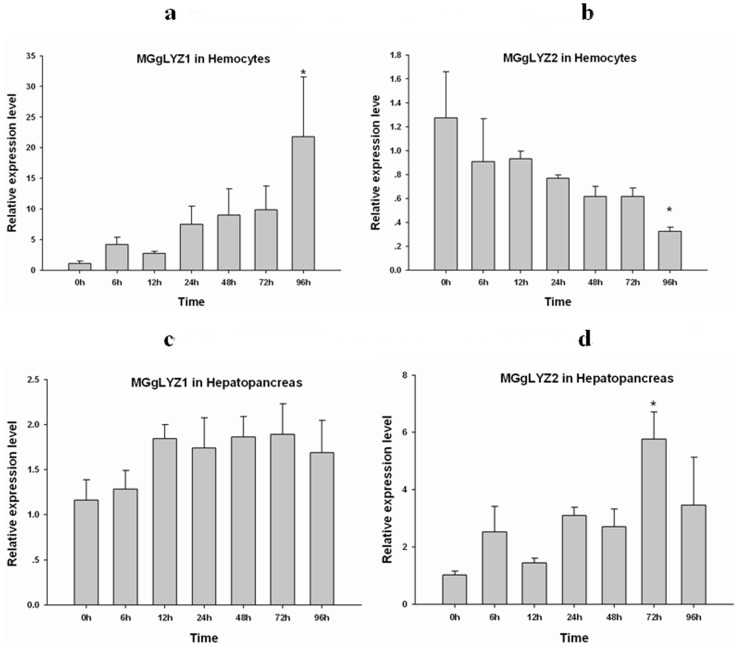
Temporal expression profiles of gLYZ mRNAs in hemocytes (a-MGgLYZ1, b-MGgLYZ2) and hepatopancreas (c-MGgLYZ1, d-MGgLYZ2) after bacterial challenge. The mRNA expression level is calculated relative to actin expression and shown as mean ± SD (n = 4). Significant difference from control (0 h) is indicated with an asterisk at *P*<0.05.

The results of Kolmogorov-Smirnov test indicated that the distributions of the data were normal and Levene's test demonstrated the variances of the data were homogeneous. After *V. anguillarum* challenge, the expression of MGgLYZ1 transcript in hemocytes (Figure 6a) was up-regulated gradually and increased up to 21.8-fold of the control group (*P*<0.05) at 96 h. However, the expression of MGgLYZ2 mRNA displayed a declining pattern after the challenge and decreased to the minimum value at 96 h (Figure 6b), which was significantly lower than that of the control (*P*<0.05). In hepatopancreas, no significant change in the expression level of MGgLYZ1 transcript was observed after bacterial challenge (Figure 6c), while the expression level of MGgLYZ2 transcript increased significantly at 72 h after challenge (Figure 6d).

### Protein recombinant, mass spectrometry identification and polyclonal antibody preparation

**Figure 7:**
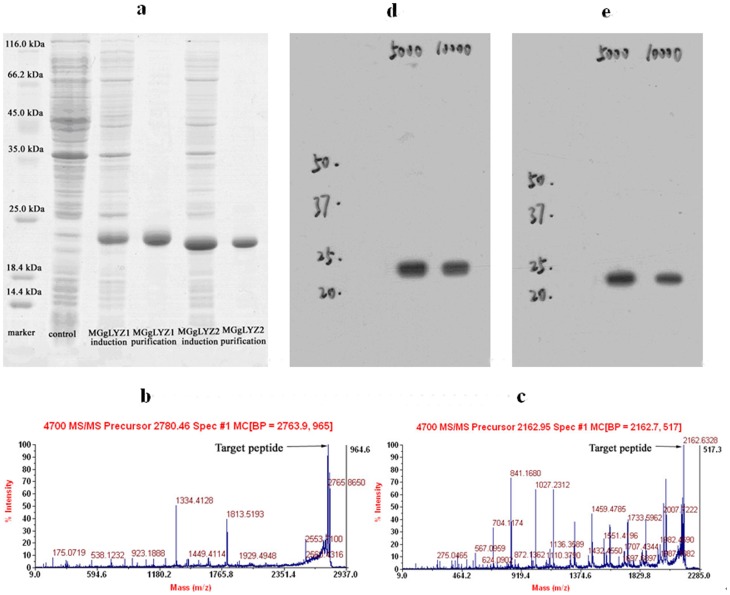
Analysis of recombinant MGgLYZ1 and MGgLYZ2 proteins and antibodies. (a) SDS-PAGE analysis, LC-ESI-MS/MS identification of MGgLYZ1 (b) and MGgLYZ2 (c), western blotting analysis of MGgLYZ1 (d) and MGgLYZ2 (e) polyclonal antibodies.

The recombinant proteins were visualized by Coomassie Bright Blue R250 staining after SDS-PAGE. The target proteins were detected with a molecular weight (MW) of about 22 and 21 kDa respectively, consistent with the expected MWs of the mature proteins (Figure 7a, lane 4 and lane 6). One of the peptide fragments (–LDIGTTHNDYSNDVIAQAQWLIHR–) identified by LC-ESI-MS/MS was identical to 163–187 of the mature peptide of MGgLYZ1 (Figure 7b), and another peptide fragment (–AQWLISHYHWHHHHHH–) corresponded to rMGgLYZ2 was also identified (Figure 7c). The recombinant proteins incubated with the corresponding antibodies (1∶5000 for MGgLYZ1, Figure 7d; 1∶10000 for MGgLYZ2, Figure 7e) showed only one band on the PVDF membrane respectively, indicating the specificity of these antibodies.

### Localization of MGgLYZ1 and MGgLYZ2 proteins

**Figure 8:**
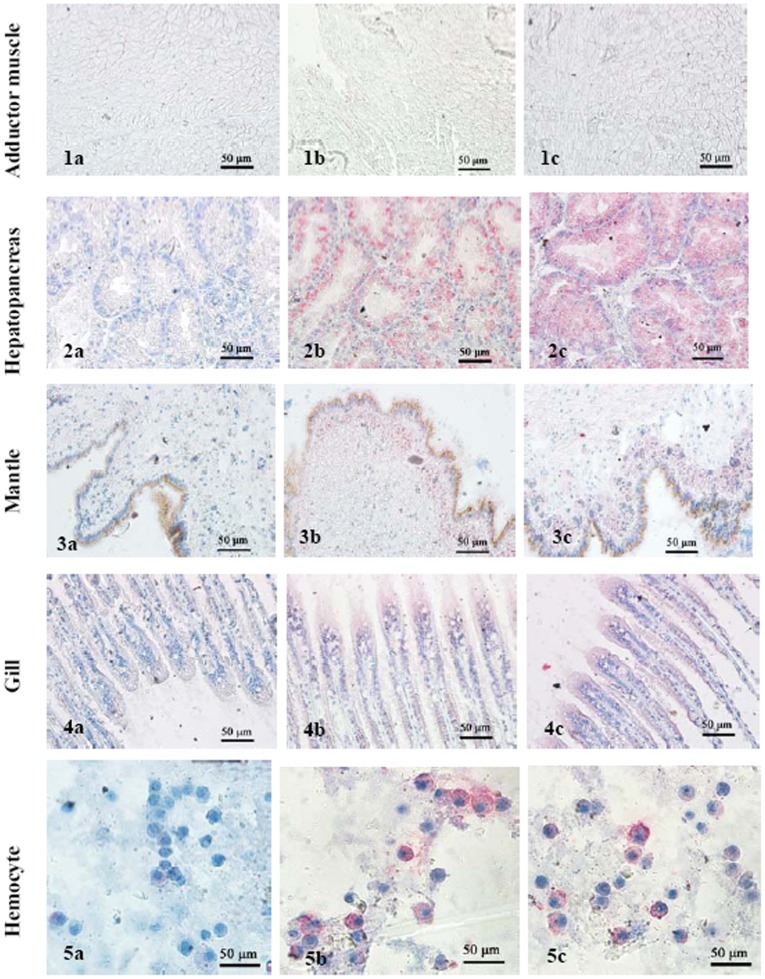
Immunolocalization of MGgLYZ1 and MGgLYZ2 in different tissues. Negative control of muscle (1a), hemocytes (2a), mantle (3a), hepatopancreas (4a) and gill (5a). Localization of MGgLYZ1 in muscle (1b), hemocytes (2b), mantle (3b), hepatopancreas (4b) and gill (5b). Localization of MGgLYZ2 in muscle (1c), hemocytes (2c), mantle (3c), hepatopancreas (4c) and gill (5c). Positive signal was stained red and the other signals were stained blue.

Immunolocalization analysis revealed similar expression pattern in hepatopancreas, gill, hemocytes and mantle with the exception of adduct muscle. In hepatopancreas, intense staining was observed in epithelia cells of the digestive tubules, but not in the connective tissues surrounding the digestive tubules (Figure 8-2b, 8-2c). In mantle, secretory cells distributed in the connective tissues were found to display weak staining (Figure 8-3b, 8-3c). In gills, immune-labeling was mainly exhibited in the columnar epithelial cells (Figure 8-4b, 8-4c). As concerned to hemocytes, strong staining was mainly detected in the granulocytes other than hyalinocytes (Figure 8-5b, 8-5c). No positive staining was detected in negative controls of different tissues (Figure 8-1a, 8-2a, 8-3a, 8-4a, 8-5a).

### Lysozyme activity profiles of rMGgLYZ1 and rMGgLYZ2

**Figure 9:**
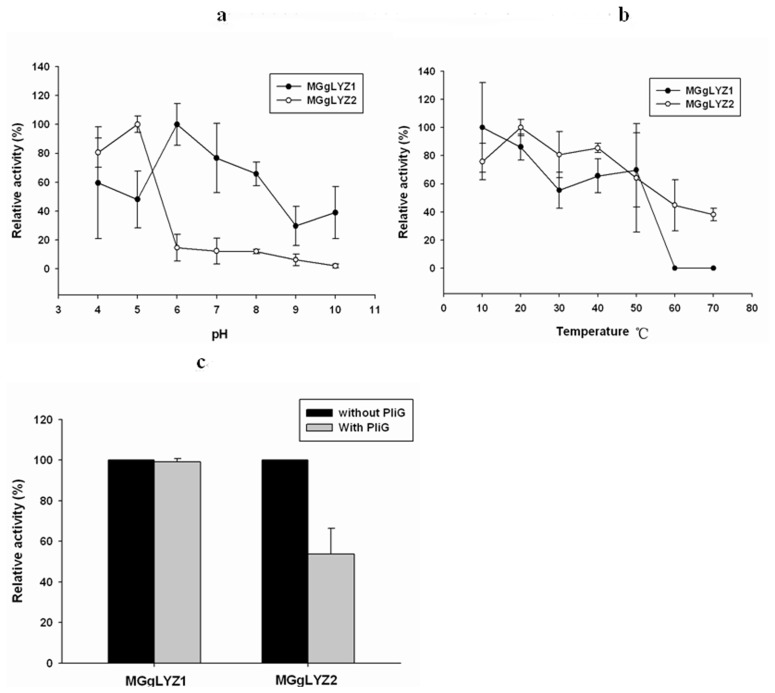
Effect of pH, temperature and lysozyme inhibitor (PliG) on the activities of MGgLYZ1 (open symbols) and MGgLYZ2 (filled symbols). Lysozyme activity is shown as % of the highest activity. The values were shown as mean ± SD (n = 3).

In this study, rMGgLYZ1 showed the highest lysozyme activity at pH 6 and relative high activity at pH 4–8 (Figure 9a). The lowest activity was detected at pH 9 with only 30% of the highest activity. As concerned to rMGgLYZ2, the maximum activity was detected at pH 5 and the activity rapidly decreased to less than 15% of the maximum activity at pHs above pH 5 (Figure 9a). The optimal temperature of rMGgLYZ1 and rMGgLYZ2 was 10°C and 20°C, respectively. Both rMGgLYZ1 and rMGgLYZ2 exhibited the lowest activity at 60–70°C (Figure 9b). The gLYZ inhibitor PliG (76 µg L^−1^) had no effect on the activity of rMGgLYZ1, while it inhibited rMGgLYZ2 activity remarkably (t-test, *P*<0.05, Figure 9c).

### MIC assay of rMGgLYZ1 and rMGgLYZ2

The antimicrobial activities of rMGgLYZ1 and rMGgLYZ2 were investigated against several Gram-positive and Gram-negative bacteria. Both rMGgLYZ1 and rMGgLYZ2 could inhibit the growth of all the tested microorganisms (Table 2), indicating that rMGgLYZ1 and rMGgLYZ2 were broad-spectrum antibacterial proteins. The highest activity of rMGgLYZ1 was found against *S. pasteuri*, *V. parahaemolyticus* and *P. putida* with the MIC of 0.95–1.91 µM, while rMGgLYZ2 had the highest activity against *P. putida* with the MIC of 1.20–2.40 µM.

**Table 2:** MIC values of recombinant MGgLYZ1 and MGgLYZ2.

Tested microorganisms	MIC value of rMGgLYZ1	MIC value of rMGgLYZ2
Gram-positive bacteria		
*Staphylococcus pasteuri*	0.95–1.91 µM	2.40–4.79 µM
*Staphylococcus aureus*	1.92–3.83 µM	2.40–4.79 µM
Gram-negative bacteria		
*Vibrio parahaemolyticus*	0.95–1.91 µM	2.40–4.79 µM
*Vibrio azureus*	1.92–3.83 µM	2.40–4.79 µM
*Enterobacter aerogenes*	1.92–3.83 µM	2.40–4.79 µM
*Enterobacter cloacae*	1.92–3.83 µM	2.40–4.79 µM
*Pseudomonas putida*	0.95–1.91 µM	1.20–2.40 µM
*Proteus mirabilis*	1.92–3.83 µM	2.40–4.79 µM

## Discussion

Goose-type lysozyme catalyzes the hydrolysis of the β-1,4-glycosidic linkage between NAM and NAG alternating sugar residues in the bacterial peptidoglycan, and causes bacterial cell lysis [7]. They were mainly found to exist in vertebrate, whereas in invertebrate, gLYZ was reported only in the scallops and gastropod [7], [24]–[25]. In this study, two gLYZs were identified from *M. galloprovincilias*. Both MGgLYZ1 and MGgLYZ2 possessed a typical gLYZ domain and the conserved residues essential for the catalytic activity and the substrate binding sites of gLYZs. Crystal structure of Atlantic cod goose-type lysozyme indicated the presence of NAG in the substrate binding sites at both sides of the catalytic residue Glu^73^. In addition, the two residues Asp^90^ and Asp^101^ in cod goose-type lysozyme were found to be involved in catalysis [39]. The multiple alignment results indicated the residues Glu^82^, Asp^97^ and Asp^108^ perhaps involved in catalysis were totally conserved in MgGLYZ1 and MgGLYZ2. Unlike c-type lysozyme, gLYZs from different animals have varied numbers of cysteine, ranging from zero to ten [22]–[23]. Most mollusk gLYZs possessed four conserved cysteines, however, the distribution pattern was different from the conserved cysteines in avians and mammals. The high content of cysteine residues in marine mollusk gLYZs was proposed to render the proteins more stable with a compacter structure in high osmolarity conditions of seawater [7], [40].

**Figure 10:**
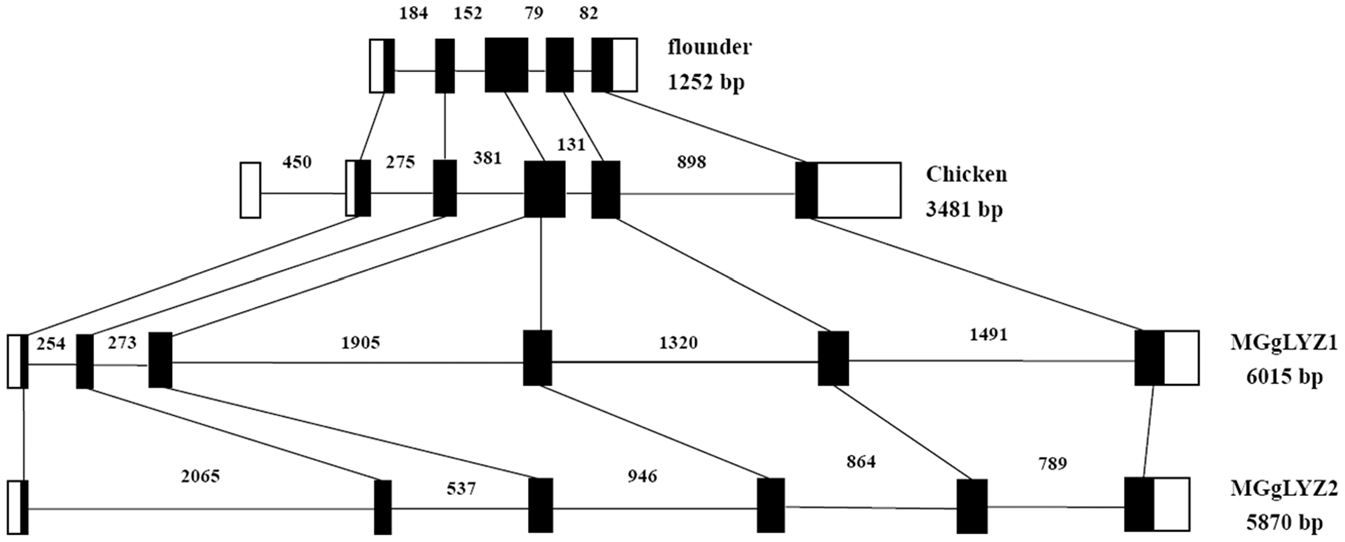
The genomic structures of MGgLYZ1 and MGgLYZ2 compared with those of Japanese flounder and chicken gLYZ genes. Black boxes indicate the corresponding open reading frames in the mussel, flounder and chicken genes. White boxes indicate the untranslated regions. The size of each intron is shown near each intron region.

To our knowledge, the genomic organization of invertebrate gLYZs has been barely investigated so far. In this study, six exons of MGgLYZ1 were found to be separated by three relatively large introns (1.3–1.9 kb) and two small introns (0.25–0.27 kb), while MGgLYZ2 were separated by five relative large introns range in size from 0.5 to 2.1 kb. Previous study had shown that the number of exons for gLYZ gene varied from five in fish to seven in human gLYZ2. Comparing the genomic structure of gLYZs from *M. galloprovincialis* and other animals (Figure 10), it could be concluded that gLYZ genes had largely maintained their genomic structure although the sizes and number of noncoding introns varied between different phyla. Therefore, the exons of gLYZ genes were thought to be evolved independently, and the lack of signal peptide in some fish gLYZs could not be ascribe to the loss of exon encoding the signal peptides during the evolution. In addition, the presence of two gLYZ genes in *M. galloprovincialis* was perhaps originated from gene duplication, for the coding exons of gLYZ genes were conversed from invertebrate to vertebrate [22].

Nevertheless, the structure of the *M. galloprovincialis* gLYZ gene differed in several ways from that of the Japanese flounder gLYZ gene. The insertion sites of introns 2, 4 and 5 of the *M. galloprovincialis* gLYZ gene corresponded with those of introns 2, 3 and 4 of the Japanese flounder gLYZ gene, respectively (Figure 10). An additional large intron 3 was inserted compared to the Japanese flounder and chicken gLYZ gene. Studies on gLYZ genomic structure in *M. galloprovincialis* and vertebrate revealed that the third and fourth exons in MGgLYZ genes were combined into a single third exon in the vertebrate genes. In this view, MGgLYZ genes appeared to have acquired more introns after the divergence of the vertebrate gLYZs. There was also another possibility that intron losses had occurred during the gLYZ evolution from invertebrate to vertebrate. Moreover, the catalytic residues Glu^82^ and Asp^97^ in exon 4 and Asp^108^ in exon 5 of the MGgLYZ genes were conserved with Glu^71^ and Asp^84^ in exon 3, and Asp^101^ in exon 4 of the Japanese flounder gLYZ gene, respectively. Repetitive elements had been identified in many lysozyme genes, such as the dispersed satellite repeats in bovine gLYZ gene and the extensive tandem repeats in the second intron of flounder gLYZ gene [2]. In this study, the dispersed satellite repeats and tandem repeat were also detected in MGgLYZ1, while only tandem repeat was found in MGgLYZ2. Interestingly, most of the MgGLYZ1 and MGgLYZ2 intron structures did not have the traditional spliceosomal intron 5′-GT donor and 3′-AG acceptor sites. Thus, the genomic structure of MGgLYZ genes was found to exhibit a different exon-intron organization pattern, and unique to all known structures of gLYZ genes.

Previous studies have demonstrated that some avian gLYZs exhibited high antibacterial activity against Gram-positive bacteria [20]–[21]. However, several fish gLYZs were reported to have antibacterial activity against both Gram-positive and Gram-negative bacteria [2], [41]–[42]. Similar to the *C. farreri* and *O. hupensis* gLYZs, both MGgLYZ1 and MGgLYZ2 displayed a lytic activity against Gram-positive and Gram-negative bacteria, implying that invertebrate gLYZs have the ability to cope with wider range of bacterial strains and species.

It had been demonstrated the higher pH was preferred for the activity of gLYZs from fish and chicken [42]–[43]. In this study, the activity of MGgLYZ1 was relatively high at the pH of 6–8. However, MGgLYZ2 exhibited high activity within a narrow acid pH of 4–5, suggesting that MGgLYZ2 might be specialized to digestive organs and acidic environments. The optimal temperature for fish [2], [39], [44]–[45] and avian [21], [46] gLYZ activities was found in the range 22–30°C and 30–40°C respectively. In the present study, both MGgLYZ1 and MGgLYZ2 exhibited high activity in the range of 10–20°C. Thus, MGgLYZs were regarded as low-temperature active enzymes to adapt the marine environment where the temperature usually ranged from 0 to 30°C.

More notably, PliG displayed obviously inhibitory effect on the enzyme activity of MGgLYZ2, whereas no effect was observed on MGgLYZ1. Vanderkelen et al. found that PliG could inhibit the activity of gLYZ from goose, salmon and larvacean [47]. The different effect of PliG on MGgLYZ1 and MGgLYZ2 might be ascribed to negative electrostatic environment in the entire substrate binding crevice and different electrostatic surface properties in their three-dimensional structure [48].

Lysozyme could be utilized as digestive enzyme to hydrolyze bacteria as one of food sources in some animals [3]. The stomach lysozymes in ruminants, leaf-eating monkeys (langur) and hoatzin had acquired the digestive function and adapted to the acid environment in the stomach. These adaptations included lowering of the isoelectric points (pI), elimination of the acid-labile Asp-Pro bond, and reduction of the number of arginine and acid-labile amino acids within the mature protein sequences [10], [49].

In this study, MGgLYZ2 possessed 11 basic amino acids (5 Arg+6 Lys), while MGgLYZ1 harbored 18 basic amino acids (7 Arg+11 Lys) which might be the cutting sites of protease. Thus, MGgLYZ2 had a lower isoelectric point and fewer protease-cutting sites than MGgLYZ1. Another common significant feature of digestive lysozymes was low net charge which brought about acidic pH optima. In mammals, the conventional lysozymes were basic proteins with net charge between +6 and +8 at pH 7, while the digestive lysozymes were almost neutral with net charge between −2 and +1 [50]. As predicted by ExPASy program, MGgLYZ1 had a net charge of +2, and MGgLYZ2 possessed a net charge of −1. Thus, MGgLYZ2 was probably more adaptive than MGgLYZ1 to acid environment because of low net charge. Furthermore, MGgLYZ2 did not have the acid-labile bond which existed in MGgLYZ1 between Asp^96^ and Pro^97^. In view of these points, MGgLYZ2 perhaps adapted to function in the acidic and protease-rich environment of the digestive gland.

Expression pattern of gLYZ genes had been investigated in several organisms. For example, fish gLYZ transcripts were mainly expressed in immune organs or organs exposed to environment, such as head kidney, spleen, blood and gill [2], [4], [42]. The transcript of scallop gLYZ was found to be mainly expressed in gills, hepatopancreas and gonad, weakly expressed in hemocytes and mantle [7]. In the present study, MGgLYZ1 mRNA was mainly expressed in the tissues exposed to the external environment, indicating the potential involvement of MGgLYZ1 in the immune responses. However, MGgLYZ2 transcript was predominantly expressed in hepatopancreas, implying that MGgLYZ2 might serve as a hydrolase against the bacteria [27].

The immunolocalization of MGgLYZ1 and MGgLYZ2 indicated a certain level of protein expression in different tissues of *M. galloprovincialis*. Both MGgLYZ1 and MGgLYZ2 were mainly expressed in epithelia cells of the digestive tubules, implying that MGgLYZs were secreted by the epithelia cells and then entered the digestive tubules to perform their function. Similar phenomenon was also found in oyster *C. gigas* and *C. virginica*, where i-type lysozyme were found to be expressed in the basophil cells of digestive tubules [51]. Additionally, both MGgLYZ1 and MGgLYZ2 were detected in granulocytes other than hyalinocytes, suggesting possible involvement of gLYZs in host immune defense. Similarly, c-type lysozyme was also found to localize within granules, especially in the large granules of *Mytilus edulis* [52]. Thus, the granules were probably the main organelles storing gLYZ in granulocytes. In gill and mantle, MGgLYZs were detected in the epithelial cells or the cells underneath the epithelia, so that these enzymes could respond quickly to the bacterial invasion. There was no gLYZ expression was detected in adductor muscle by immunolocalization analysis. However, low expression level of gLYZ mRNA was detected by real-time PCR in the adductor muscle, which was perhaps originated from the contamination of hemocytes retained in this tissue.

It had been widely demonstrated that gLYZ transcription was enhanced by bacterial challenge, particularly in immune relevant organs of fishes [2], [4], [41]. Until recently, the mRNA expressions of three snail gLYZs were found to be significantly increased responding to parasite infection [25]. In the present study, the expression of MGgLYZ1 transcript in hemocytes was significantly increased after *Vibrio* challenge, indicating the involvement of MGgLYZ1 in innate defense responses. However, the down-regulation of MGgLYZ2 mRNA in hemocytes suggested that MGgLYZ2 perhaps played a much less role in immune defense. As concerned to hepatopancreas, the transcriptional level of MGgLYZ2 was significantly up-regulated after bacterial challenge, but no change was found in MGgLYZ1 expression level. These results indicated MGgLYZ2 might perform its function mainly in the hepatopancreas and play a significant role in digestion.

Several studies had reported that the digestive lysozymes in ruminants had evolved under positive selection pressure [13], [17], [49], [53]–[54]. Six residues were found to undergo adaptive changes for digestive lysozyme function in ruminants and colobine monkeys, including Lys/Glu^14^, Lys/Glu^21^, Glu^50^, Asp^75^, Asn^87^ and Lys/Glu^126^. In oyster *C. virginica*, i-type lysozyme also had an episode of positive selection associated with the functional transition from defense to digestion. However, positive selected sites were not calculated [55]. In the present study, three positive sites in the mature peptides of MGgLYZ2 were detected by M2a and M8 model. The Tyr^102^ residue located at the second β-strand of the secondary structure, which was vital for the structure stability of MGgLYZ2 [56]. The other two positively selected amino acids Leu^200^ and Ser^202^ situated on the fifth α-helix of MGgLYZ2. According to the predicted three-dimensional structure, the fifth α-helix of MGgLYZ2 was much longer than that of MGgLYZ1, which perhaps increased the hydrophobicity of MGgLYZ2. In addition, Ser^202^, as a neutral amino acid, was more adaptive to acid environment than the basic amino acid His^202^ in MGgLYZ1. In view of these points, the increased hydrophobicity may be conductive to the adaption of MGgLYZ2 to acidic environment.

In conclusion, two gLYZ were identified from *M. galloprovincialis*, and their molecular characteristics, enzymatic properties, spatial and temporal expression profiles were also investigated. It was found that MGgLYZ1 was involved in host defense and MgGLY2 mainly served as a digestive enzyme in hepatopancreas. Further analysis indicated the evolution of MGgLYZ2 was obviously under positive selection. Our findings showed for the first time that mollusk goose-type lysozymes underwent adaptive evolution from self-defense to digestion.

## Supporting Information

Figure S1 **The genomic structures of MGgLYZ1 (a, 6015 bp) and MGgLYZ2 (b, 5870 bp).** Exon coding regions are indicated by capital letters and protein sequences coded by exons are shown by one capital letter below the nucleotide sequence. Introns and untranslated regions (underlined) are shown in lower case letters.
(DOCX)

Figure S2 **A Bayesian phylogenetic tree of mollusk gLYZs.** Numbers at the forks indicate Bayesian posterior probabilities. The sequences of mollusk gLYZs are listed in Table S3.
(TIF)

Table S1 Primers used for gene structure identification in this study.
(DOCX)

Table S2 Primers used for qRT-PCR, recombination and polymorphism detection.
(DOCX)

Table S3 The sequences used to construct phylogeny trees of gLYZs.
(DOCX)
