# Supplementary material for: Two Goose-Type Lysozymes in Mytilus galloprovincialis: Possible Function Diversification and Adaptive Evolution
Source: PLoS One. 2012 Sep 21;7(9):e45148. doi: 10.1371/journal.pone.0045148 (PMC3448621; doi:10.1371/journal.pone.0045148)
Supplement: Figure S1 — The genomic structures of MGgLYZ1 (a, 6015 bp) and MGgLYZ2 (b, 5870 bp). Exon coding regions are indicated by capital letters and protein sequences coded by exons are shown by one capital letter below the nucleotide sequence. Introns and untranslated regions (underlined) are shown in lower case letters. (DOCX) [file pone.0045148.s001.docx]

**Figure S1**

**a Nucleotide sequences of MGgLYZ1 gene—6015 bp**

**1 acaacaagatttcttacagaggagaaatccattctggtttccctcatcggaggtatcaac
     61 acaatttgaagaacactaattgttgtcagttcagctgataattctcagaagttaattttc
      1  M  E  N  I  L  V  V  L  A  V  L  V  S  V  E  A             
    121 aATGGAAAATATTTTGGTAGTTCTTGCTGTATTGGTATCAGTTGAAGCAataggtaggta
    181 tagctaattgatagccgttttaggcatgtgttttttatatgtttatttgaattcatggtt
    241 ttcaacaataaaagcttatttaaaaatgtgaaactttgaaatcattaactttatcgagca
    301 gtaaactttgataattataattcttgtcgtaagtcgaccgttagatattcaaaaactaac
    361 agtaaataacttttatacactcgagtaagtattggataattaacgtcaatgcttttgtgt
     17    I  D  Y  N  C  H  G  N  V  T  V  L  H  P  K  G  M  A  P  
    421 ttcATAGATTATAACTGCCATGGTAACGTGACAGTTCTACATCCTAAAGGCATGGCTCCT
     36 K  Y  G                                                     
    481 AAATACGGTggtaagaaccttgctacaaatcattaggctaaacccctgtatgagtcagtg
    541 gttaatgatgctgagacttattgtatttacttaacgttcaatggaaactatttgaacatt
    601 aactttccaattaaagattttcatatttatgtcctctccaaaatcaaccaaacaagcatc
    661 acatgcatttttttaattgatcagatagtaaatctcaggactgtgttgatttttttatgt
     39                                           G  M  A  A  S  H  
    721 agattaaattttacatttaattgtctttcactttgtattgcaGGAATGGCAGCCTCCCAT
     45 L  A  I  D  Q  D  I  S  E  I  D  K  R  K  S  C  Y  L  K  A  
    781 CTCGCCATAGACCAGGATATAAGTGAAATAGATAAACGAAAGTCCTGTTATCTTAAAGCA
     65 A  A  N  N                                                  
    841 GCAGCTAATAACtgtgagtcatattcaatttattgcatggcgcaattttctattaatttt
    901 gtttaatctttgtaagttatttgagccaattgaaccttcatctgaccggtgtaacattaa
    961 agctgctttacatattttcttctgtccaatttacaatgcatcgacagatgtagcctttat
   1021 ttaagtgttcaattttaattatacctatcgagcaaatgaactgttgatttgaaattaaaa
   1081 agacatgttacaaggacaaatagttacaacataaactttttactaaacaactacattgta
   1141 cgtatgtttttagcaaaaacagaatattttaggcactatttaaagttaaaagcaaggaat
   1201 catgtattttaaagggtcctcgctagtgtttgatatgcccttcgctgttcaaatttttat
   1261 attaaatttcccccgtgtatatatgtaaactcaatgttaccgtagttggtaccagttcaa
   1321 aggacagtcgttcagtcaactatatttaatgaatggctattatttattttgaatttattg
   1381 aaccataaaatgaagttttgactcttcacattgaataatcggcgaatcataaaaaattga
   1441 caacattgaaaataaaactaatacttttaaccaatcagaagacagtaaatatatcaaatt
   1501 tatttatatcgttatgaaactagatagatgaacactggattataaatcctgtagaaatgt
   1561 cacctcaagggtgaccgtggaatgatagtatggtcactattattcttttttgaccagcag
   1621 ttaaatccttgccgaaatggggagtctgttggactgtgcggaatgcacagatacgaattc
   1681 aagtctaatcataatggggacattatatccgatggtatatacaccgtgatgtagagtaag
   1741 tgccactctatcttcacatgagaaacatactacatttttaaggggtccgttggttgcctg
   1801 ttacaaggtaaaaatttccggccctattcaattcaatataacgtccttttctagtggcag
   1861 tacgaccttcatcacagatagcttctattacagaacctgcctgttgcatttcttgttgtt
   1921 ttactctcgccctaaacatgtaatattcgacactgaatattaagcaatcaaaaatcaatc
   1981 ttgtcatataattttggcgaaaaattatcacaacgttaatatcataatgtatgtacaaaa
   2041 ttaatgtggttacaaatttatggatattcttttctttggaaaagaacatgtaaaataatc
   2101 taaaaaataagtaaatacgtaatacaaacagaaaaatgtaatcagaaacgaagaaacaga
   2161 tttagattcgagaagagtaaactatatattaggaggtcggatacgctgagtaagatttca
   2221 tacatcttaaatcaaattttcgaatataggttctaggtgaaaccgattgatggtgctgct
   2281 gcaaaaataagtaatctcgtttcctttggaaatttaaaaatagttgttttaacagaatat
   2341 cgaatatgtaaaaatggacctacttatgaacggacattccaaaaaggggggtgggaagga
   2401 tatgttcgaaataaaaattatagaggtctagatgagggtctttattgccattttcttaaa
   2461 tcgttaggtttgctttgtagactctttacgtctccctcccttttcataatctctgttcgc
   2521 gagtctacaatgcaacgttaaaatattatcgccaagtacatgtgtgcatttggcttcctc
   2581 agacgtcattgattctatcattcaaaactgaaatagcgcatgttaacaaaaacttgtcgc
   2641 taagccgtatatctgtagtaagtgtgtagtatatagtactaaatgatattcaaactgaaa
     69                                                          C  
   2701 ctcttaaaaactattaaagttagaatacaactttcgtccctatgcgtcatcattgtaTGC
     70 I  H  P  A  V  I  A  G  I  A  S  R  E  S  R  A  G  K  M  L  
   2761 ATACACCCAGCTGTGATTGCCGGTATTGCTAGCAGAGAGTCTCGTGCTGGAAAGATGTTG
     90 Y  S  T  N  G  W  A  D  H  H  H  A  Y  G  I  M  Q           
   2821 TACAGTACCAATGGTTGGGCTGATCATCACCATGCTTATGGCATCATGCAGgtatatact
   2881 atgtaaacacttaaattcattttggtaaaacacgtgcaaagatgatttaaagcagactgc
   2941 tcagctcggagtcagaatttcgtgttcgagtagggtgccttcttattgtggaatgttacg
   3001 gactagcactttataaatccggtctaacgtgtcaatctaatacaactcatggtcacattc
   3061 atacatgtcaaggattccattcacatgtttctcgtcctgaatatgcatttgatataggta
   3121 aaaccaccattaatcaacatcatcatccttaacaatacgaacgtttaattggatggtgaa
   3181 atttgcatggtgtttcgtgaaaacaatacatttaaatcgacgatatcggagccataatac
   3241 aaatgtctgaattttgataaaaaggatgtaatacctgtttatatcacctatagatattta
   3301 atgaaaagtgtttcagaaattttcagatataaaaataggaagatgtggtatataaatgca
   3361 aaagagacaactatcccctagcgacaaaatgacgcgtaaacatatagaaaactccatgtc
   3421 accgtacggccatcagaaatgaacaaacatataccgtatagtcagctacaaatgtataat
   3481 agcaacgtacgtacgacatgtacttacataaaacatttattttaccaaaaaataataata
   3541 ttgtccataactttacaactacagacatttatagggggtctcattgggggtttctgatcc
   3601 cggatcccgcttactgttttgccagattcccgtatcccgcttactgttttgtcagattcc
   3661 cgtatcccgcttacactatgtacgtaagcaattctcgtttttttttgtcatttcccgggt
   3721 cccgctagacctcatttcatgttttcacgacacaataatttgactttcgcgtgtcacgct
   3781 tacaaaaaatcggcaatcccgagtcacgcttagaccccaatgagacccactttatactgc
   3841 aactagttgtgtttatttcctaaatatagtaccacagctatnattttngtgcaatgtcaa
   3901 tttcatcatggaacacttttccacagntcagggattcattaagggatgaaaataagtttg
   3961 aaatttgtataancaatttaaatagctatgaatttatttatttaagtttcagtataaaaa
   4021 aaatatagtgtcaatgtcagaaaaatatcaactttttgtactcggcgcaaaactggggaa
   4081 ggtctcggtagaacctggcccttccccagtttctcagcctcatacaaaaaagttgacatt
    107                                                    C  D  V  
   4141 tttctgacatttacctaatattgtatatttattatgcgagtttaatttcagTGTGATGTC
    110 R  V  D  P  L  H  P  Y  H  K  N  C  T  S  Y  L  W  Y  S  C  
   4201 CGAGTCGATCCGCTTCACCCTTACCACAAAAACTGCACATCTTACCTCTGGTACAGCTGT
    130 D  H  I  N  A  M  T  K  Y  V  L  V  P  Y  I  E  A  V  K  Q  
   4261 GATCATATCAACGCCATGACAAAATATGTCTTAGTACCATACATAGAGGCTGTGAAACAA
    150 K  L  P  S  W  S  D  A  Q  A  P  Q                          
   4321 AAACTACCATCATGGTCGGATGCTCAAGCACCACAAggtattaatagcaataactgcatt
   4381 tgattataaaccatcataaataaaaggtcaacaataaatactatataccatatactaagt
   4441 atttcttaatattgataaaaatgaaaagcagacacataatggaaactcaaactacaagta
   4501 caaaagaaaaacagcaacagtacacgttttatcacaattagacggattttatatgtcaat
   4561 tgataacgttgtacatgtatatttcggatacaattaatgcatgatatatgttttagtgta
   4621 tataattaaaacatatatcgactgagctgtttattcatatatccgtaatacctaaattaa
   4681 tatataatagtttactgtattgcaggaacccaaactaggttcgagggagttaaattcctg
   4741 aaacgaaaacaatttctcatgttttatttcaatttgaactcatattgcatatttattttc
   4801 aaaaattctttctaggatcgatgatatcatcggggacttatagtcaaccaccagcaatta
   4861 ctatagaaccacattcaacaaacgatatttttcgtatgctcgaggctatcagtttcgaaa
   4921 tgcacattgcattaaaaaaaattcccacaacctttaaattattcagaccttgtatttaga
   4981 aataatttacaaattaacccgatctctattcaatgaagttatcaattttgtatcaataaa
   5041 agtaaatgtatgataaacgtcaacgagacggcaacacaacgatacttttttttctatttg
   5101 tttggccattataaatgcatttgtaaatatttgtgtattaatgttatgaatggaaatatt
   5161 tcaatctacggtataatatttatttatttggcgtcacacaattcgtacgtgtatatatgt
   5221 aggaagatgtggtatgagtgtcaatgagacaactctccatccaagtaacaatctataaaa
   5281 gtaaaccattataggtcaaggtacggccttcaacacggagccttggctcacaaacgaaca
   5341 gcaagctataaagggcccccaaaaaatactggtgtaaaaccattcaaacgggaaaaccaa
   5401 cggtataatctatataaaaacgagaaacgagaaacaattatgaaccacataaacagacga
   5461 caaccactgaacatgcatataattatgaaattgactcttaatctcaaaactaccttgtaa
   5521 aacacaagatatagtttctaaaaatcaagtattcataaagaaaaagtggatagaaatgga
   5581 aacaaaagaaacaaatatattgactgttttatttgattatttataaaaagttatcagcaa
   5641 aaatactggacactactactcgcataagtaaccatcaacacccgactactaaaatctagg
   5701 atgtaccaacaaatacaaataacaaccagcttccttgacttaatccagacaaaaaggatg
   5761 gtctggttaaatattgtttcacgtgatcacaaaccttacccttttcccatatagagcgtt
    162                            G  G  V  A  A  Y  N  F  G  V  R  
   5821 cgtcgtaacaccattttatattctttaGGTGGAGTGGCAGCATACAATTTTGGAGTACGC
    173 N  V  R  T  W  D  K  L  D  I  G  T  T  H  N  D  Y  S  N  D  
   5881 AATGTTAGGACCTGGGATAAATTAGATATTGGAACAACGCATAATGATTATAGTAATGAC
    193 V  I  A  Q  A  Q  W  L  I  H  R  Y  N  W  *
   5941 GTAATTGCACAGGCCCAGTGGTTAATTCATCGTTATAATTGGTAGaatccgaatatttaa
   6001 aacagttcaaataaa**

**b Nucleotide sequences of MGgLYZ2 gene——5870 bp**

**1             M  K  T  F  F  L  L  F  A  V  I  F  A  T  D  A  
      1 tactgtgaagcaATGAAAACGTTTTTCCTTTTATTTGCTGTAATTTTTGCAACTGATGCA
     61 gcgagtaagtaaataagtttttgaaatattccttttaaatgtattttataatacatttat
    121 ggaaatttatataagcatgtattttataatacatttatggaaatttatataagcatttat
    181 taatgaaattttagaaaaatgaagcattccctaaataagacaatcatcaaatgttttttt
    241 cggtatatcataccatttaactacaaataccagtattcctcgtgtgtaattttggagtag
    301 atgtgtatattctacgaatgcagaaaaccattttatattttctagatcgtagatttttta
    361 tgaacaataatcaacatgtacaattattcgatatcaactattatggttttatcgtggtat
    421 ttcgtatgttacggcagtgttaaaggtcttcggaagtctaggttatctttacatttgatt
    481 ttgaaacgaaattatattcagataacatcaagcttaatttgccgtctacctgtgatagtg
    541 cgtgatatttgaaaaatagaaaaacacacaaatgaaacacagtaaaattaaagattcatg
    601 ttttaagcgcaatcgcattggtcactatgctacgtttgtatgtctgctagtattgaatta
    661 ttctgacaagtgtatgattggtcttgatgggtttatgatacaggatgtaacgttataaaa
    721 tcaaagatgtcttattttagattattccataatgtattatagcattcattatatagcagc
    781 gagctcgtcttgatgttgcctacatatttgggttttggtggtttgagtagttggagtggt
    841 ccgatttactgtaaaacactagtttgtgattgctgtaatcctttttgttcaatatgttaa
    901 tcttttacggtaatataaataaataaattatgctgtttgtttgttttaacaatgttgcaa
    961 gtgtaaagttaaaatataggaaacgaggggaaaacaattcttgaaatttgaaaataatac
   1021 agtcaaagcgtgtacacttggccgtttcagattgtaaaggcctatagataagttcattgt
   1081 ttgtaccttaaaatgaaaaaagggacacaatttgttgaatttccattgggtatgatgatt
   1141 ttaaccgatcaccacgctttggtgtacactttggaaaatataacctagcatgcattatat
   1201 tcgaataaggcgaactgcaaaacacgagtattacaacagaggcacaaaacacttaacatt
   1261 ttttgtcctcttattcagaatgtgacctaccgaattatacttttaccgggttaatattta
   1321 catgagcaacacgacgagtatcacatatgaagcaggttctatttacccgtccggatcatc
   1381 tgatatcactccagtatttgattgggttcgtgttggtcagtcttaagttgtatgcgtggt
   1441 gtaatgtgtactgttgtttgtctgttgaacactttcttttctgagacatgactttgtcag
   1501 tttattttcgacttatgaaatcttttgttgctgtttaacatgtttgttttcctttcactg
   1561 tttgatacatgaattaggtcgttagttctacgtttgtcattttgggaccttttatagctg
   1621 actgagcggtatgaactttgtactttgctgaaggccgtgtaatgacttgtattttttcat
   1681 ttcggtgtcattttatctcttgggaagtgttcctaatggcaatcttcttatttttataat
   1741 gttaaacataaatgaaaatataatttgaatgttaccttatttcaaactgggaagatcgtg
   1801 ataaaagctataaaaaaggagcatagtcttcaaaacaaaatgtttgtatgctccaatact
   1861 tacaagtattatcttcaaatgataaaacaattcaaacgaatggacaacaactgtcctatt
   1921 ggtacatgcattttcatgcaaaatgtagaaaatggttgattgaacatagaagacccgtaa
   1981 tgcttttgtataagatatgataatgtttgtgatattatgcgatatgttgaaagctttaat
   2041 cacaatttaaatcttgttaatatttgattttatttttttaacatgacgaaaatatataag
     17                          A  N  Y  N  C  H  G  D  V  T  Q  L 
   2101 aatattatatgaccaaattttatatGCGAACTATAATTGTCATGGTGATGTGACACAGCT
     29  H  P  T  G  M  G  S  A  Y  G                               
   2161 TCATCCAACAGGAATGGGCAGTGCATATGGAagatatcaatgatataataaaacaatccc
   2221 tgttgcgaagcagagacaacgttaccaatatttcggagtgtcgatatgaataaatcagag
   2281 attttgtagaagccaattggatagggacaagacaatactaaaaaaaatatcttatcaatc
   2341 ctataacaaaataattttcgaataatatgaaataaaataaatgtgttaatcgatcaccaa
   2401 tttcaattatctcgttcgttttaagcaaatccaatagggcgttaatcaatgtgtcttttt
   2461 tcgaaatctagagactttattacctttgtttgcaaatattgaccactgaaaataaggaac
   2521 attttttttttagattttatggaaagattaacaaattgaaagcaacccatgacaattaaa
   2581 ttacttgaacataaaacaagttcaaattcttgtttgtgaatcacttttgataaaatacaa
   2641 agtctccgattcagtattttttttctttttttttggagaaaactacgcataatttgatta
     39                             G  M  A  G  S  H  Q  A  I  D  Q 
   2701 tgtaaaatatgttttgtgtgtttaatagGGTATGGCAGGTTCGCACCAGGCTATTGATCA
     50  D  I  A  E  I  N  K  R  K  S  C  Y  V  Q  A  G  A  A  N    
   2761 GGATATTGCTGAGATCAATAAACGAAAGTCTTGTTATGTACAAGCAGGAGCTGCGAACtg
   2821 taagtatttatattgatatctattttagtataccctcccgattgtttcatttttttctgt
   2881 ttctatgtagtaatactacatacgctcgcagaacaattttgattctaagaaattataaaa
   2941 agataaaaagttaaattacaaaaatactgaactccgaggaaaattcaaaacggaaagttc
   3001 ttcatcaaatagcaaaatcaaatgataaaacacatcaaacgaatggacaacaactgtcat
   3061 attcctgacttggtacagaaattttcaaatgtaaaatattatatataagtatcagcttgg
   3121 attaaaacaatctgcgactatctttttatgtcgtttttcgttgttgtttttctaaactca
   3181 acattgtatgttgaactagctgtattttcattcttcaagtagtatgatcaaaacaaaaga
   3241 tatcttgtttcaatgcaattcaaactcactcttagagttcttatttcggaaatctaagtt
   3301 ctggcttggttggtttgcaattcttgcgcatatgtgcaacaacatatatatttttagctt
   3361 gtaggaagacaagttttactgctcctatttccttttatagttttatgctttaagcttaaa
   3421 tattttatgctctggtgaataattctttgagtccgattttattattccggaaagtcattt
   3481 ggaaacggatgtaatactcatgttcacgttgacatttttataactttcgtattgtcaaac
   3541 aattattttatttttaaattcaaactcactattaaagttcttatttgggaaacgatgaac
   3601 taaagtgtattacttgcaaatattgctcgttttttatgctgcgataaaaactctttcaag
   3661 gttttttttataaagtcgggattgagattttgaaacgaatatgatattcatgttttcttt
     69                                             C  I  H  P  A  V
   3721 ttaaattttgataacttccataatgtcgaatgattattttatagTGTATACATCCTGCAG
     75   I  A  G  L  A  S  R  E  S  R  A  G  K  L  L  Y  S  T  S  G
   3781 TTATTGCTGGTCTTGCTAGTCGGGAATCTCGCGCCGGAAAGCTTTTATATAGTACAAGCG
     95   W  G  D  H  H  N  A  Y  G  I  M  Q                        
   3841 GCTGGGGTGATCATCATAACGCATACGGTATCATGCAGgtagacagaatatgaaatcaat
   3901 ggatttgcttacttccatttatgtattactcttttccaaaatacaccgctatttgctgct
   3961 gatgagtaacaaatcagctttgcaatactcagtcattatttttttttaaattaatactag
   4021 ggtaatgaattatactatagtcagaaatttacttgatgttttggaacaagtattatgtat
   4081 ctgattctttttttagaaagggaaggctgcaaacgtacgaaaggtcaattagtgtcatca
   4141 tttaaaagtgcttataataatacatgtatgaatatgcaattaacaagtagaattgcagga
   4201 taaatgtggctcttttgtaatgaaaacgggtatgtaagcagtcaggtaatgaccagcacg
   4261 tcacaaaacggattgcataagaatatatacaatgtttgtaccttttgtaatactcaccgc
   4321 gtattctgaatttcatgaataggtaatgaatatatctataaacaaagatataattttttt
   4381 tatgaaaatgtgatgagattttacttattttagatgctatgttgtctttaaaaaattaat
   4441 tcggagtgttttgaatatatataaaaaatcaagaaacaaaataataagaataagtcagaa
   4501 ttatattttcgttgtcttattgttgcaaaatctacgagtctaactaaggaacttcagtag
   4561 aagtataaacctgtcaaaacaatggtaaccaaactaggctcgtaggttccttgctattta   4621 ctggtttggttcatttgttgtttttctgttttgtctaggatctaagggtaacagtaatca
   4681 atgctaacataattcatttttaaaactgtatgcattatttgattgacttattttcattcc
    107   C  D  I  N  A  N  P  L  H  S  I  H  K  T  C  T  S  Y  H  W
   4741 agTGTGACATCAATGCTAACCCACTACATAGTATTCATAAAACATGTACATCTTACCACT
    127   D  S  C  A  H  I  N  A  M  T  A  H  V  L  V  P  N  I  Q  G
   4801 GGGACAGCTGTGCCCACATAAACGCTATGACAGCGCATGTCCTAGTTCCAAATATACAAG
    147   V  K  R  K  H  H  S  W  S  D  A  Q  A  L  Q               
   4861 GTGTTAAACGCAAGCATCATTCGTGGTCGGATGCGCAAGCGTTACAAggtttgtcatcat
   4921 ataaaatagatagttccctttctgttacattgtaccggtagctttatacaattattaaca
   4981 aatggtgtaggtctaatatatagcataacgaatcatgttatgctatcatgaagacctgca
   5041 aagaatgtttaaataatatttacaaatttagatggacataaactgccatacatgttataa
   5101 ttctttaacagtttataaaacaatgtcaccaaatcaaaggatttatagggaaataaaatg
   5161 aaatacttgttgactcacaatgtacagcagaaggtcaccaacaggtcttcaatgtagata
   5221 gaaattcccgcacccggaggcgtgcttcagctggcccttaaaaaaaatatactagttcag
   5281 tgataatgaacgccatactaatttccaaattgtacacaagaaactaaaattaaaataatt
   5341 caagactaacaaaggccaaaggatcctgacttgggacaggcgcaaaaatgcggcggggtt
   5401 aaacatgtttatgagatctcaaccctcttcctatacctctagccaatgtagaaaagtaaa
   5461 acacacttctcactttttaaataacacacaacactagttaattttcaaataaggtacttc
   5521 ctaccaatgtgaatacaaaatgacattataagtgcttcagttttggaatgtctggtataa
   5581 acaatcagacaacatctttttagaaagaaaaaaacttgtatggggacgttttttcgcagt
    162                                                         G  G
   5641 aacctgaagattattataattatgtaacacnttttacatttgtattttcctttttaGGTG
    164   V  A  A  Y  N  F  G  L  G  N  V  Q  S  W  G  G  L  D  V  G
   5701 GAGTAGCTGCATACAACTTTGGTTTAGGCAATGTACAGAGTTGGGGCGGATTGGATGTGG
    184   S  T  H  N  D  Y  S  N  D  V  I  A  R  A  Q  W  L  I  S  H
   5761 GATCAACACATAATGACTACAGCAACGACGTAATTGCACGTGCTCAATGGCTAATTAGCC
    204   Y  H  W  *
   5821 ATTATCATTGGTAGtatgtagaagggtcatcctttaaacacacaaataaa**
