# Supplementary material for: Two Goose-Type Lysozymes in Mytilus galloprovincialis: Possible Function Diversification and Adaptive Evolution
Source: PLoS One. 2012 Sep 21;7(9):e45148. doi: 10.1371/journal.pone.0045148 (PMC3448621; doi:10.1371/journal.pone.0045148)
Supplement: Table S1 — Primers used for gene structure identification in this study. (DOCX) [file pone.0045148.s003.docx]

Table S1. Primers used for gene structure identification in this study

| Primer | Sequence (5’-3’) | Sequence information | Location at the gene |
| --- | --- | --- | --- |
| P1 (forward) | TGGTAGTTCTTGCTGTATTGGT | Primer for MGgLYZ1 intron 1 | 135-156 |
| P2 (reverse) | ATGCCTTTAGGATGTAGAACTG | Primer for MGgLYZ1 intron 1 | 452-473 |
| P3 (forward) | TACATCCTAAAGGCATGGCTCC | Primer for MGgLYZ1 intron 2 | 458-479 |
| P4 (reverse) | TATCCTGGTCTATGGCGAGATG | Primer for MGgLYZ1 intron 2 | 778-799 |
| P5 (forward) | CCATCTCGCCATAGACCAGGAT | Primer for MGgLYZ1 intron 3 | 777-798 |
| P6 (reverse) | CTGTACAACATCTTTCCAGCACG | Primer for MGgLYZ1 intron 3 | 2803-2825 |
| P7 (forward) | GTCTCGTGCTGGAAAGATGTTG | Primer for MGgLYZ1 intron 4 | 2799-2820 |
| P8 (reverse) | TGTTTCACAGCCTCTATGTATGG | Primer for MGgLYZ1 intron 4 | 4297-4319 |
| P9 (forward) | CCATACATAGAGGCTGTGAAAC | Primer for MGgLYZ1 intron 5 | 4297-4318 |
| P10 (reverse) | AGGTCCTAACATTGCGTACTCC | Primer for MGgLYZ1 intron 5 | 5872-5893 |
| P11 (forward) | GCTGTAATTTTTGCAACTGATGC | Primer for MGgLYZ2 intron 1 | 37-59 |
| P12 (reverse) | ATTCCTGTTGGATGAAGCTGTG | Primer for MGgLYZ2 intron 1 | 2154-2175 |
| P13 (forward) | TTCATCCAACAGGAATGGGCAG | Primer for MGgLYZ2 intron 2 | 2160-2181 |
| P14 (reverse) | ATCAATAGCCTGGTGCGAACCT | Primer for MGgLYZ2 intron 2 | 2737-2758 |
| P15 (forward) | AGGTTCGCACCAGGCTATTG | Primer for MGgLYZ2 intron 3 | 2737-2756 |
| P16 (reverse) | AGATTCCCGACTAGCAAGACC | Primer for MGgLYZ2 intron 3 | 3789-3809 |
| P17 (forward) | CTGGTCTTGCTAGTCGGGAATC | Primer for MGgLYZ2 intron 4 | 3787-3808 |
| P18 (reverse) | CTGTCATAGCGTTTATGTGGGC | Primer for MGgLYZ2 intron 4 | 4812-4833 |
| P19 (forward) | AACGCAAGCATCATTCGTGGTC | Primer for MGgLYZ2 intron 5 | 4867-4888 |
| P20 (reverse) | GTGTTGATCCCACATCCAATCC | Primer for MGgLYZ2 intron 5 | 5748-5769 |
