# Supplementary material for: Two Goose-Type Lysozymes in Mytilus galloprovincialis: Possible Function Diversification and Adaptive Evolution
Source: PLoS One. 2012 Sep 21;7(9):e45148. doi: 10.1371/journal.pone.0045148 (PMC3448621; doi:10.1371/journal.pone.0045148)
Supplement: Table S2 — Primers used for qRT-PCR, recombination and polymorphism detection. (DOCX) [file pone.0045148.s004.docx]

Table S2. Primers used for qRT-PCR, recombination and polymorphism detection

| Primer | Sequence (5’-3’) | Sequence information |
| --- | --- | --- |
| P21 (forward) | TGTAACAAACTGGGACGATA | Real time primer forβ-actin |
| P22 (reverse) | AGCATGAGGAAGGGCATAAC | Real time primer forβ-actin |
| P23 (forward) | ATCCTAAAGGCATGGCTCCT | Real time primer forMGgLYZ1 |
| P24 (reverse) | TAGCAATACCGGCAATCACA | Real time primer forMGgLYZ1 |
| P25 (forward) | TGCCCACATAAACGCTATGA | Real time primer for MGgLYZ2 |
| P26 (reverse) | GTTGATCCCACATCCAATCC | Real time primer for MGgLYZ2 |
| P27 (forward) | CATATGATAGATTATAACTGCCATGG | Recombinant primer for MGgLYZ1 |
| P28 (reverse) | CTCGAGCTAGTGGTGGTGGTGGTGGTGCCAATTATAACGATGAA | Recombinant primer for MGgLYZ1 |
| P29 (forward) | CATATGGCGAACTATAATTGTCATGG | Recombinant primer for MGgLYZ2 |
| P30 (reverse) | CTCGAGCTAGTGGTGGTGGTGGTGGTGCCAATGATAATGGCTAA | Recombinant primer for MGgLYZ2 |
| P31 (forward) | CCATTCTGGTTTCCCTCATC | Primer for MGgLYZ1 polymorphism |
| P32 (reverse) | CGATGAATTAACCACTGGGC | Primer for MGgLYZ1 polymorphism |
| P33 (forward) | TACGTCGAATACTGTGAAGCA | Primer for MGgLYZ2 polymorphism |
| P34 (reverse) | GACCCTTCTACATACTACCA | Primer for MGgLYZ2 polymorphism |
