# Supplementary material for: Two Goose-Type Lysozymes in Mytilus galloprovincialis: Possible Function Diversification and Adaptive Evolution
Source: PLoS One. 2012 Sep 21;7(9):e45148. doi: 10.1371/journal.pone.0045148 (PMC3448621; doi:10.1371/journal.pone.0045148)
Supplement: Table S3 — The sequences used to construct phylogeny trees of gLYZs. (DOCX) [file pone.0045148.s005.docx]

Table S3. The sequences used to construct phylogeny trees of gLYZs

| Species | Taxonomy | Accession numbers |
| --- | --- | --- |
| *Homo sapiens* | Vertebrate: Mammalia | AAI00886 |
| *Mus musculus* | Vertebrate: Mammalia | AAI47568.1 |
| *Gallus gallus* | Vertebrate: Aves | NP_001001470 |
| *Anser anser anser* | Vertebrate: Aves | P00718 |
| *Cygnus atratus* | Vertebrate: Aves | P00717 |
| *Rhea Americana* | Vertebrate: Aves | JC7955 |
| *Casuarius casuarius* | Vertebrate: Aves | Q7LZR3 |
| *Struthio camelus* | Vertebrate: Aves | BAL03620.1 |
| *Dromaius novaehollandiae* | Vertebrate: Aves | BAL03618.1 |
| *Danio rerio* | Vertebrate: Teleostei | AAH76099 |
| *Esox lucius* | Vertebrate: Teleostei | ACO13238.1 |
| *Osmerus mordax* | Vertebrate: Teleostei | BT075322.1 |
| *Oncorhynchus mykiss* | Vertebrate: Teleostei | ACO08249.1 |
| *Salmo salar* | Vertebrate: Teleostei | ACI69019.1 |
| *Ictalurus furcatus* | Vertebrate: Teleostei | ADO28271.1 |
| *Ambystoma mexicanum* | Vertebrate: Amphibia | AEQ98812.1 |
| *Xenopus laevis* | Vertebrate: Amphibia | NP_001088153.1 |
| *Oikopleura dioica* 1 | Urochordata | CAD92342 |
| *Ciona intestinalis* | Urochordata | XP_002122566.1 |
| *Branchiostoma floridae* | Cephalochordata | XP_002611412.1 |
| *Mytilus galloprovincialis* 1 | Invertebrate: Mollusca | JQ244770 |
| *Mytilus galloprovincialis* 2 | Invertebrate: Mollusca | JQ244771 |
| *Chlamys farreri* | Invertebrate: Mollusca | DQ227696.1 |
| *Argopecten irradians* | Invertebrate: Mollusca | AY788903 |
| *Mizuhopecten yessoensis* | Invertebrate: Mollusca | GR867752.1 |
| *Mytilus californianus* | Invertebrate: Mollusca | ES392226.1 |
| *Lottia gigantean* | Invertebrate: Mollusca | FC670738.1 |
| *Nesiohelix samarangae* | Invertebrate: Mollusca | DC603639.1 |
| *Biomphalaria glabrata* | Invertebrate: Mollusca | ES491677.1 |
| *Tritonia diomedea* | Invertebrate: Mollusca | EV289297.1 |
| *Physella acuta* | Invertebrate: Mollusca | ADV36303.1 |
| *Ilyanassa obsolete* | Invertebrate: Mollusca | FK716269.1 |
| *Oncomelania hupensis* 1 | Invertebrate: Mollusca | GW425811 |
| *Oncomelania hupensis* 2 | Invertebrate: Mollusca | GW426148 |
| *Oncomelania hupensis* 3 | Invertebrate: Mollusca | GW427036 |
